# The gas-phase formation mechanism of iodic acid as an atmospheric aerosol source

**DOI:** 10.1038/s41557-022-01067-z

**Published:** 2022-11-14

**Authors:** Henning Finkenzeller, Siddharth Iyer, Xu-Cheng He, Mario Simon, Theodore K. Koenig, Christopher F. Lee, Rashid Valiev, Victoria Hofbauer, Antonio Amorim, Rima Baalbaki, Andrea Baccarini, Lisa Beck, David M. Bell, Lucía Caudillo, Dexian Chen, Randall Chiu, Biwu Chu, Lubna Dada, Jonathan Duplissy, Martin Heinritzi, Deniz Kemppainen, Changhyuk Kim, Jordan Krechmer, Andreas Kürten, Alexandr Kvashnin, Houssni Lamkaddam, Chuan Ping Lee, Katrianne Lehtipalo, Zijun Li, Vladimir Makhmutov, Hanna E. Manninen, Guillaume Marie, Ruby Marten, Roy L. Mauldin, Bernhard Mentler, Tatjana Müller, Tuukka Petäjä, Maxim Philippov, Ananth Ranjithkumar, Birte Rörup, Jiali Shen, Dominik Stolzenburg, Christian Tauber, Yee Jun Tham, António Tomé, Miguel Vazquez-Pufleau, Andrea C. Wagner, Dongyu S. Wang, Mingyi Wang, Yonghong Wang, Stefan K. Weber, Wei Nie, Yusheng Wu, Mao Xiao, Qing Ye, Marcel Zauner-Wieczorek, Armin Hansel, Urs Baltensperger, Jérome Brioude, Joachim Curtius, Neil M. Donahue, Imad El Haddad, Richard C. Flagan, Markku Kulmala, Jasper Kirkby, Mikko Sipilä, Douglas R. Worsnop, Theo Kurten, Matti Rissanen, Rainer Volkamer

**Affiliations:** 1 Department of Chemistry, University of Colorado Boulder, Boulder, CO USA; 2 Cooperative Institute for Research in Environmental Sciences, University of Colorado Boulder, Boulder, CO USA; 3 Aerosol Physics Laboratory, Physics Unit, Faculty of Engineering and Natural Sciences, Tampere University, Tampere, Finland; 4 Institute for Atmospheric and Earth System Research, University of Helsinki, Helsinki, Finland; 5 Institute for Atmospheric and Environmental Sciences, Goethe University Frankfurt, Frankfurt, Germany; 6 State Key Joint Laboratory of Environmental Simulation and Pollution Control, BIC-ESAT and IJRC, College of Environmental Sciences and Engineering, Peking University, Beijing, China; 7 Department of Chemistry, University of Helsinki, Helsinki, Finland; 8 Center for Atmospheric Particle Studies, Carnegie Mellon University, Pittsburgh, PA USA; 9 CENTRA and Faculdade de Ciências da Universidade de Lisboa, Lisboa, Portugal; 10 Laboratory of Atmospheric Chemistry, Paul Scherrer Institute, Villigen, Switzerland; 11 Extreme Environments Research Laboratory, École Polytechnique Fédérale de Lausanne, Lausanne, Switzerland; 12 Research Center for Eco-Environmental Sciences, Chinese Academy of Science, Beijing, China; 13 Helsinki Institute of Physics (HIP) / Physics, Faculty of Science, University of Helsinki, Helsinki, Finland; 14 School of Civil and Environmental Engineering, Pusan National University, Busan, Republic of Korea; 15 Division of Chemistry and Chemical Engineering, California Institute of Technology, Pasadena, CA USA; 16 Aerodyne Research, Billerica, MA USA; 17 P.N. Lebedev Physical Institute of the Russian Academy of Sciences, Moscow, Russia; 18 Finnish Meteorological Institute, Helsinki, Finland; 19 Department of Applied Physics, University of Eastern Finland, Kuopio, Finland; 20 Moscow Institute of Physics and Technology (National Research University), Moscow, Russia; 21 CERN, the European Organization for Nuclear Research, Geneva, Switzerland; 22 Institute of Ion and Applied Physics, University of Innsbruck, Innsbruck, Austria; 23 School of Earth and Environment, University of Leeds, Leeds, UK; 24 Faculty of Physics, University of Vienna, Vienna, Austria; 25 School of Marine Sciences, Sun Yat-sen University, Zhuhai, China; 26 IDL-Universidade da Beira Interior, Covilhã, Portugal; 27 Joint International Research Laboratory of Atmospheric and Earth System Research, School of Atmospheric Sciences, Nanjing University, Nanjing, China; 28 LACy UMR8105, Université de la Réunion, Saint-Denis, France; 29 Aerosol and Haze Laboratory, Beijing Advanced Innovation Center for Soft Matter Sciences and Engineering, Beijing University of Chemical Technology (BUCT), Beijing, China

**Keywords:** Atmospheric chemistry, Photochemistry, Physical chemistry

## Abstract

Iodine is a reactive trace element in atmospheric chemistry that destroys ozone and nucleates particles. Iodine emissions have tripled since 1950 and are projected to keep increasing with rising O_3_ surface concentrations. Although iodic acid (HIO_3_) is widespread and forms particles more efficiently than sulfuric acid, its gas-phase formation mechanism remains unresolved. Here, in CLOUD atmospheric simulation chamber experiments that generate iodine radicals at atmospherically relevant rates, we show that iodooxy hypoiodite, IOIO, is efficiently converted into HIO_3_ via reactions (R1) IOIO + O_3_ → IOIO_4_ and (R2) IOIO_4_ + H_2_O → HIO_3_ + HOI + ^(1)^O_2_. The laboratory-derived reaction rate coefficients are corroborated by theory and shown to explain field observations of daytime HIO_3_ in the remote lower free troposphere. The mechanism provides a missing link between iodine sources and particle formation. Because particulate iodate is readily reduced, recycling iodine back into the gas phase, our results suggest a catalytic role of iodine in aerosol formation.

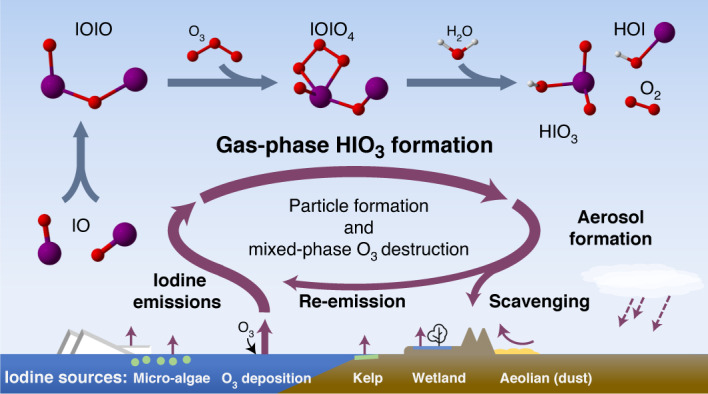

## Abstract (web summary)

Iodic acid (HIO_3_) forms aerosols very efficiently, but its gas-phase formation mechanism is not well understood. Atmospheric simulation chamber experiments, quantum chemical calculations and kinetic modelling have now revealed that HIO_3_ forms as an early iodine oxidation product from hypoiodite. The mechanism explains field measurements and suggests a catalytic role for iodine in particle formation.

## Main

Iodine is a trace constituent of the atmosphere that is particularly efficient at forming new particles. While sulfuric acid (H_2_SO_4_)^1–3^, methanesulfonic acid^1,4^ and nitric acid^5^ all require an additional vapour (ammonia, NH_3_ or dimethylamine (DMA)) to form particles, highly oxygenated organic molecules (HOMs)^6^ and iodine^7–9^ can do so alone. Iodine nucleation rates exceed those of H_2_SO_4_ (in excess NH_3_) at comparable concentrations of iodic acid (HIO_3_)^10^. Furthermore, HIO_3_ growth rates of nanoparticles are both charge- and dipole-enhanced, exceeding the neutral collision rate^10,11^.

Currently, iodine particle formation is rarely represented in atmospheric models—such models form most particles from the nucleation of H_2_SO_4_ and include iodine primarily because of its ozone-destroying potential^12^. While sulfur emissions are projected to decrease due to pollution control measures (probably to a few tens of teragrams of SO_2_ per year by 2100 (ref. ^13^), iodine emissions have been increasing due to human activity. Iodine is primarily emitted from oceans by the reaction of O_3_ with iodide (I^−^) dissolved in surface waters, which liberates volatile iodine species (hypoiodous acid (HOI) and iodine (I_2_)) to the atmosphere^14,15^. This marine source is enhanced as a result of O_3_ pollution on local and hemispheric scales^16,17^ as well as the thinning of sea ice^18^, and now accounts for iodine emissions of ~3 Tg yr^−1^ (refs. ^19,20^). Over the past 70 years, iodine concentrations have tripled in ice-core records in Greenland^18^, Alpine glaciers^17^ and tree-ring records in Tibet^21^.

Iodine is highly reactive and participates in catalytic reaction cycles that enhance its atmospheric impact. A catalytic role is well known for O_3_ loss, but has, as of yet, not been suggested for particle formation. Iodine in the lower stratosphere has a 6–15 and 400–1,000 times higher O_3_ destruction potential per atom than bromine and chlorine^22^. Extremely low mixing ratios of iodine oxide (IO) radicals (for example, ~0.1 parts per trillion by volume (pptv); IO = 10^−13^ volume mixing ratio) can therefore affect the lifetime of climate-active gases (for example, O_3_ and CH_4_)^19,23,24^. This chemical reactivity extends to heterogeneous reactions involving aerosol iodide (I^−^)^14,15^ and iodate (IO_3_^−^) (refs. ^25,26^ and references therein), which is the thermodynamically most stable form of iodine. The efficient multiphase chemistry of IO_3_^−^ is markedly different from that of inert aerosol sulfate (SO_4_^2−^), which accumulates without further chemical conversion until it is scavenged from the atmosphere by wet or dry deposition.

Iodine is ubiquitous in the atmosphere^22,23,27,28^, and HIO_3_ has been detected in coastal marine air^9,10,29^, the Arctic and Antarctic boundary layer^9,10,30–32^, various continental sites^10^ and in the lower free troposphere^10,33^. Several precursors for HIO_3_ have been suggested: hydrated iodine atoms^10,34^, hydrated IO radicals^34^, iodine dioxide (OIO) radicals^35^ and larger iodine oxides (I_2_O_3_, I_2_O_4_ and I_2_O_5_; refs. ^34,36–38^). However, these mechanisms remain speculative and have not been demonstrated experimentally, leaving atmospheric HIO_3_ observations unexplained. Recent field observations of iodine-induced nucleation over remote oceans^31^ and of IO_3_^−^ in stratospheric aerosols^22^ suggest a widespread role of iodine particle formation, but the conundrum of the missing HIO_3_ source mechanism blocks our ability to connect iodine sources to particle formation in atmospheric models.

## Results and discussion

### CLOUD measurements

In this Article we report iodine chemistry and particle formation experiments under marine boundary layer conditions at the CERN CLOUD chamber (Methods). Because of the large chamber volume (26.1 m^3^) and associated long wall-loss lifetime (~8 min; comparable to typical condensation rates in the atmosphere), precursor gas-phase concentrations do not need to be increased above atmospheric levels (Supplementary Table 1). Experiments were conducted at 283 K and 263 K, with I_2_ at a typical volume mixing ratio of 8 pptv (range of <0.5–330 pptv), 40% relative humidity (RH, <3–90%) and 40 ppbv O_3_ (<1–80 parts per billion by volume (ppbv)). The chemistry is driven by photolysis of I_2_, which is measured by cavity-enhanced differential optical absorption spectroscopy (CE-DOAS; Methods) and bromide chemical ionization mass spectrometry (Br^−^-MION-CIMS). HIO_3_ is measured quantitatively by NO_3_^−^-CIMS, and HOI by Br^−^-MION-CIMS. Both instruments also allow insights into the evolution of other iodine species (IO, OIO, I_2_O_2_, I_2_O_4_ and so on; Methods).

**Fig. 1:**
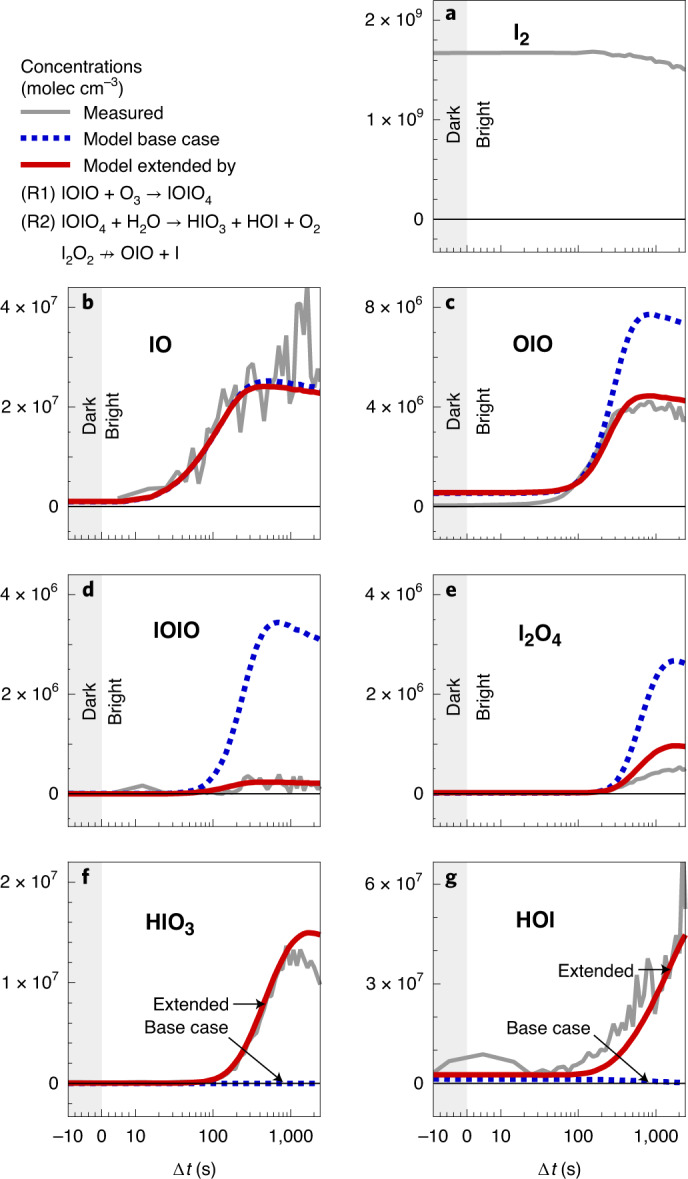
Coincident formation of HIO_3_ and HOI in the early stages of iodine oxidation. **a**–**g**, Time-resolved measurements of key iodine species (**a**,**b**,**d** show precursors to HIO_3_ (**f**) and HOI (**g**), and **c** and **e** show higher-oxide routes) are compared with model predictions after the start of I_2_ photolysis at green wavelengths within the CERN CLOUD chamber. Measured concentrations (grey lines) of HIO_3_ and HOI exceed 10^7^ molecules per cm^3^ (molec cm^−3^) within minutes. Established gas-phase iodine chemistry (model base case, dashed blue lines) forms neither HIO_3_ nor HOI, contrary to the observations, and overestimates the concentrations of IOIO and I_2_O_4_. The extended model (solid red line), including reactions (R1) and (R2) and considering a longer thermal lifetime of IOIO, achieves good mass and temporal closure for HIO_3_, HOI, IOIO and I_2_O_4_. Source data

The measurements are accompanied by chemical box modelling, building on state-of-the-art iodine chemistry (Methods). The model is constrained by measurements of I_2_ concentrations, actinic fluxes, temperature, humidity and losses of molecules to the chamber walls (stainless steel, characterized via H_2_SO_4_) and chamber dilution (~2 h). Established iodine chemistry only contains a single reaction predicted from theory^35^ that could form HIO_3_ from OIO + OH. This reaction does not form HIO_3_ in the HO_*x*_-free conditions when I_2_ is photolysed by green light^10^. Even if OH radicals were present, they would be predominately scavenged by other species. The model base case does not form any HIO_3_ or HOI under the experimental conditions probed (Fig. 1). Based on the comprehensive experimental evidence of this work, and supported by theoretical calculations, the base case model is extended to include the following two reactions:

R1 $${{{\rm{IOIO}}}}+{{{{\rm{O}}}}}_{3}\to {{{{\rm{IOIO}}}}}_{4}$$

R2 $${{{{\rm{IOIO}}}}}_{4}+{{{{\rm{H}}}}}_{2}{{{\rm{O}}}}\to {{{{\rm{HIO}}}}}_{3}+{{{\rm{HOI}}}}{+}^{(1)}{{{{\rm{O}}}}}_{2}$$

and considers an update to the thermal lifetime of IOIO (extended model, Supplementary Section 3).

**Extended Data Fig. 1:**
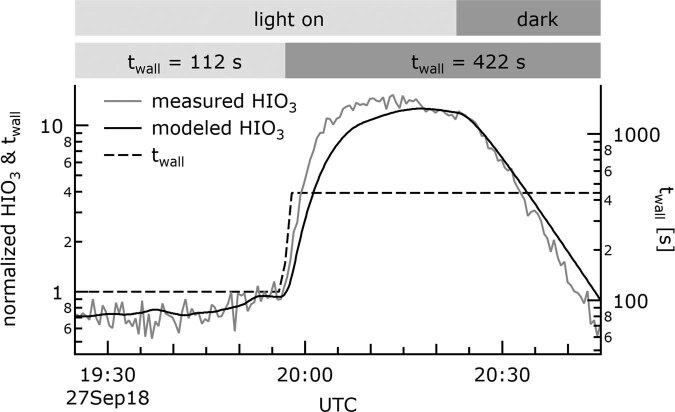
Response in the HIO_3_ concentration to varying the mixing fan speed. A strong sensitivity of the HIO_3_ concentration to changes in the wall loss lifetime t_wall_ (dashed black line) is observed. While other parameters are held constant, stirring of the CLOUD atmospheric simulation chamber is reduced at 19:57 UTC, increasing the wall loss lifetime by a factor of four. HIO_3_ concentrations recover by a factor 12. The superlinear response is evidence for a reasonably long-lived precursor (that is, IO) that gets lost to the chamber walls. At 20:25 UTC, light is turned off, HIO_3_ production stops, and the HIO_3_ concentration is efficiently lost to the chamber walls. The model reproduces the observed behaviour if IO is considered to efficiently get lost to the chamber wall. Source data

**Extended Data Fig. 2:**
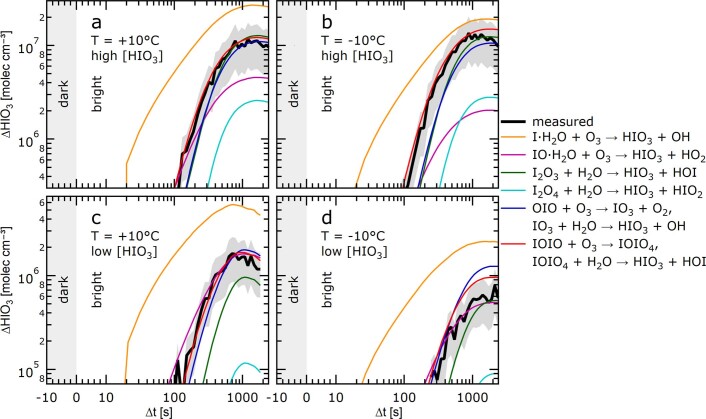
Time and mass closure of hypothetical HIO_3_ formation mechanisms. Sensitivity studies assume hypothetical mechanisms that form HIO_3_ from different precursors in the model. After the start of I_2_ photolysis (Δt = 0), ΔHIO_3_ is defined as HIO_3_(Δt) - HIO_3_(Δt = 0). HIO_3_ measurements (thick black line, grey shading indicates 50 % uncertainty) and simulated time profiles assuming different hypothesised mechanisms in the model (coloured thin lines). The four panels a-d show the closure at different temperatures, and HIO_3_ concentrations. The formation of HIO_3_ via reactions R1 and R2 is the only mechanism compatible with observations regarding temporal and mass closure. Source data

Figure 1 shows that HIO_3_ and HOI concentrations rapidly increase to exceed 1 × 10^7^ molecules per cm^3^ (molec cm^−3^) within a few minutes of the onset of I_2_ photolysis by green light (grey lines). While zero HIO_3_ and zero HOI are predicted by the base case model (current state of the art), the extended model achieves excellent agreement with regard to the measured concentrations and the timing of HIO_3_ and HOI formation. The extended model also improves the closure of timing and concentrations measured for OIO, IOIO and I_2_O_4_. Measured HIO_3_ concentrations reach a steady state after ~8 min, consistent with the wall-loss lifetime of other sticky molecules^3^ measured at CLOUD (Extended Data Fig. 1). HOI continues to accumulate due to a lower effective wall uptake. Notably, the IO radical concentrations closely resemble those in the remote marine boundary layer (compare Supplementary Table 1) and do not exceed 1 pptv (1 pptv = 2.68 × 10^7^ molec cm^−3^ at 273 K and 1 atm pressure). The timing of IO radicals is predicted very well from both the base case and the extended model, reflecting the high level of trust in the gas-phase chemical kinetics during the early stages of the iodine photolysis experiments. Interestingly, iodine oxide clusters I_*x*_O_*y*_ (*x* ≥ 2, *y* ≥ 3) larger than IOIO are formed too late to explain the rapid formation of HIO_3_ as an early generation product (Extended Data Fig. 2 and Supplementary Table 2).

**Fig. 2:**
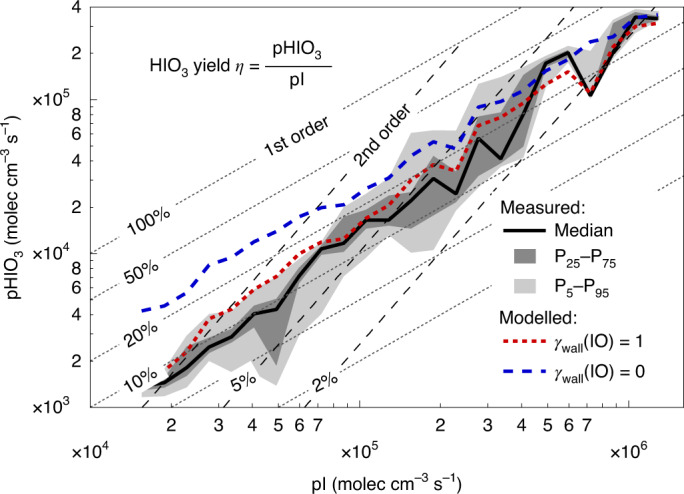
HIO_3_ yield *η* and rate order. The HIO_3_ production rate pHIO_3_ scales in first order with the I atom production rate pI (median (solid line) and 25–75% and 5–95% inter-percentile ranges (dark and light grey shading)). The yield *η* is substantial (~20%) and near constant for pI larger than 10^5^ molec cm^−3^ s^−1^. At smaller pI, losses of intermediates to chamber walls reduce *η*. This effect is captured by the model (red line (median)) and is explained by IO radical wall losses (compare blue dashed and red dotted lines (medians)). If larger I_*x*_O_*y*_ clusters were the HIO_3_ precursor, a higher-order yield would be expected—this is not consistent with the observations. Source data

Figure 2 shows that the extended model accurately predicts the measured HIO_3_ production rates, pHIO_3_, over a wide range of I radical production rates, pI (10^4^–10^6^ molec cm^−3^ s^−1^). Here, pHIO_3_ is calculated from HIO_3_ concentration measurements and the well-known loss rates to the chamber walls, and pI is calculated from the photolysis of I_2_. The HIO_3_ yield, defined as the ratio of pHIO_3_ and pI, is a function of the experimental conditions and varies between 10 and 20%. This variability is most pronounced for low pI (<10^5^ molec cm^−3^ s^−1^) and is quantitatively explained by the wall loss of HIO_3_ precursors becoming progressively more relevant at lower gas concentrations. We corroborated that HIO_3_ formation from I atoms is a multistep process by carrying out an experiment with enhanced stirring (by two fans at the top and bottom of the chamber), thereby decreasing the wall accommodation lifetime of HIO_3_ from the standard ~8 min to ~2 min, while holding all other parameters constant. The HIO_3_ concentration decreased by more than one order of magnitude, indicating that the HIO_3_ suppression exceeds that expected from a change in lifetime alone (Extended Data Fig. 1). The extended model reproduces this superlinear response under the reasonable assumption of efficient reactive uptake of IO radicals on the chamber walls (red dashed line, Fig. 2). Indeed, if the extended model is run while disregarding IO wall loss (blue dashed line, Fig. 2), a constant and high yield of ~20% applies over the full pI range probed.

**Extended Data Fig. 3:**
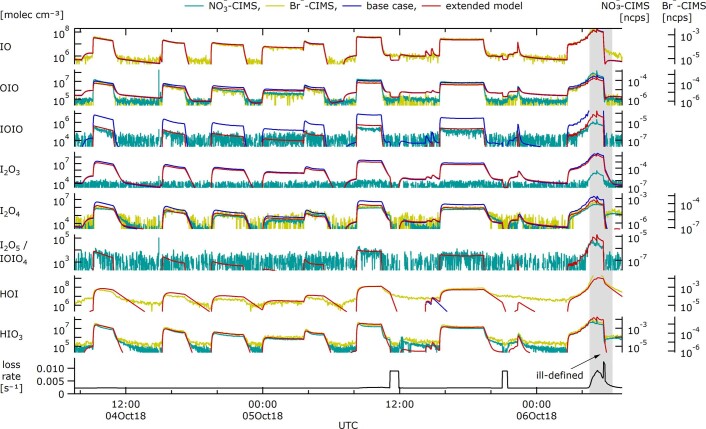
Detection of iodine oxide radicals and I_x_O_y_ species, including the key species IOIO, IOIO_4_, HOI, and HIO_3_. Concentrations of iodine species as measured by the ${{{{\rm{NO}}}}}_{3}^{-}$-CIMS and the Br^−^-MION-CIMS, and as modelled by the base-case and extended model. The bottom panel shows the loss rate of sticky molecules to the chamber walls, to particle surfaces, and to dilution. The grey shaded period shows an experiment with extremely high IO_x_ concentrations, where IOIO_4_ is clearly detected, but extreme particle concentrations and chamber inhomogeneities lead to higher model-measurement differences. The base-case model does not form any HOI or HIO_3_ in UV-dark conditions. The extended model reproduces both and improves the closure also for other molecules. Calibration factors are given in Supplementary Table 3. T = 263 K. Source data

That HIO_3_ formation is first order in pI (Fig. 2) explains the presence of HIO_3_ over remote oceans, where pI is low (Supplementary Table 1)^10,31^. This finding also carries key mechanistic information, in that it is incompatible with the hypothesis that larger I_*x*_O_*y*_ (*x* ≥ 3) species are HIO_3_ precursors^34^ at CLOUD. If such I_*x*_O_*y*_ were the precursor, the HIO_3_ yield would not be constant, but would increase progressively with pI, and pHIO_3_ would follow a higher-order rate law (Supplementary Fig. 2). This is not observed. We regularly detect I_2_O_2_ and I_2_O_4_, in agreement with predictions by the extended model (Fig. 1 and Extended Data Fig. 3), indicating that there is no fundamental limitation to our analytical capabilities to detect I_*x*_O_*y*_ species. Interestingly, I_2_O_3_ is generally not detected, except in experiments that employ extremely high I_2_ concentrations (ppbv levels), which can bias reaction pathways to favour the formation of larger I_*x*_O_*y*_ species (Supplementary Table 1 and Supplementary Section 5). Quantum chemical calculations support that the ${{{{\rm{I}}}}}_{2}{{{{\rm{O}}}}}_{3}\cdot {{{{{\rm{NO}}}}}_{3}}^{-}$ cluster is thermally stable (Supplementary Fig. 3) and should be observable. Including the formation of HIO_3_ from IOIO in the extended model reduces the predicted I_2_O_3_ by approximately a factor of two (Extended Data Fig. 3), and improves predictions about IOIO, in close agreement with observations (Fig. 1). The remaining discrepancy for I_2_O_3_ reflects the uncertainty in larger I_*x*_O_*y*_ chemistry^39^. We conclude that I_*x*_O_*y*_ species larger than IOIO are not needed as precursors for HIO_3_ under typical conditions at CLOUD.

**Extended Data Fig. 4:**
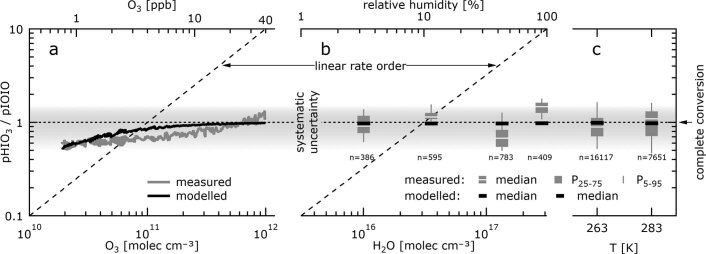
Sensitivity studies of the HIO_3_ production towards changes in O_3_, H_2_O, and temperature. For the ranges probed there is no pronounced sensitivity of HIO_3_ production (normalised by IOIO production) observed. The linear rate order lines (long dashes) assume either O_3_ or H_2_O were controlling the rate limiting step towards HIO_3_ formation. No such dependence is observed. The robustness in HIO_3_ formation is evidence that neither O_3_ nor H_2_O (nor temperature) control the rate limiting step under the conditions probed. Measurements and predictions of the extended model agree within uncertainties. Measurements: 5-95 % whiskers, 25-75 % boxes, median. Model: median only. The grey shading indicates the combined measurement model uncertainty (65 %, 2 − *σ* standard deviation). Source data

HIO_3_ formation from IOIO is robust against variations in O_3_, H_2_O and temperature (Extended Data Fig. 4 and Supplementary Fig. 1). This suggests that neither O_3_ nor H_2_O are rate-limiting to HIO_3_ formation under the conditions probed. The rate-limiting step is the formation of IOIO, which is fully converted into HIO_3_ (Extended Data Fig. 4). We observe excellent closure between pHIO_3_ and pIOIO during the O_3_ ramps, where pIOIO is based on the well-known IO + IO rate coefficients^40^.

At O_3_ concentrations below a few ppbv, the chemistry slows down sufficiently that other sinks become relevant for IOIO (for example, wall loss and thermal decomposition), resulting in a slight dependence of the pHIO_3_-to-pIOIO ratio on O_3_. That slight dependence is captured by the extended model (assuming an IOIO wall uptake coefficient *γ*_wall_(IOIO) = 1). In contrast, a pronounced O_3_ sensitivity would be expected if IO⋅H_2_O or OIO were HIO_3_ precursors (Supplementary Fig. 1). The absence of an O_3_ and H_2_O sensitivity is difficult to reconcile with any mechanism that does not quantitatively convert a single precursor. The comprehensive evidence (Supplementary Table 2 and Supplementary Section 2) strongly supports a rapid and quantitative conversion of IOIO into HIO_3_ and HOI.

### Quantum chemical calculations

We employed quantum chemical calculations (density functional theory (DFT) methods M062X/aug-cc-pVTZ-PP, followed by coupled-cluster single-point energy corrections; Methods) to explore the reactivity of IOIO with O_3_, H_2_O and other available reactants to form HIO_3_ and HOI. IOIO reacts reasonably quickly with O_3_ to form HIO_3_, HOI and singlet oxygen via reaction sequences (R1) and (R2).

**Fig. 3:**
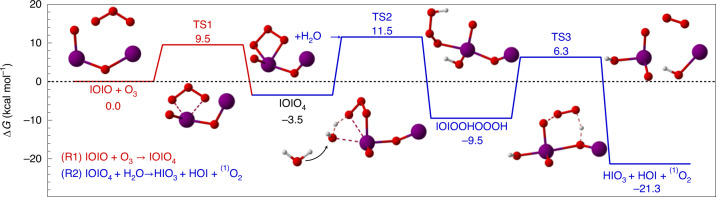
Quantum chemical calculations support HIO_3_ and HOI as co-products of hypoiodide IOIO oxidation. Reaction coordinate for the gas-phase reactions (R1) and (R2) as free energy Δ*G*(*T* = 298 K). The energies are calculated using theory at the CCSD(T)/CBS(T,Q)//M062X/aug-cc-pVTZ-PP level of theory. Δ*G*(TS3) (not rate-limiting) is calculated at the CCSD(T)/aug-cc-pVTZ-PP//M062X/aug-cc-pVTZ-PP level, due to memory limitations. The reaction coordinate supports that atmospheric concentrations of O_3_ and H_2_O lead to a quantitative conversion of IOIO into HIO_3_, HOI and singlet O_2_.

Figure 3 shows the reaction coordinate. The reactions (R1) and (R2) are exothermic and free of prohibitively large barriers. Accurately predicting energies and rate coefficients for iodine is challenging because of the inherent complexity of iodine atoms (atom size, number of electrons and relativistic effects). The strong sensitivity towards varying levels of theory is illustrated by comparing bond dissociation energies (BDEs) and proton affinities for simple iodine oxides where measurements are available (Table 1). The method used in this study has improved skill in the coupled-cluster part of the calculations, primarily due to a more balanced description of the basis set on iodine and the other atoms (Methods), and is found to reproduce experimental values within ~3 kcal mol^−1^ (with the exception of the OIO BDE), which translates into approximately one order of magnitude uncertainty in rate constants.

**Table 1:** Comparison of different levels of theory with experimental values

Reaction	Parameter	Unit	Theory^a^ (literature)	Theory^b^ (this study)	Experiment
IO → I + O(^3^P)	BDE	kcal mol^−1^	71.6	59.4	57.4^e^
OIO → IO(^2^Π) + O(^3^P)	BDE	kcal mol^−1^	81.5	64.8	58.0^e^
HI → H^+^ + I^−^	Enthalpy of deprotonation	kcal mol^−1^	356.6	316.3	314.3^f^
HOI → H^+^ + IO^−^	Enthalpy of deprotonation	kcal mol^−1^	368.5	354.4	355.6^f^
IOIO → OIO + I	*t*_therm_ (298 K)	s	1.4^c^	4.0 × 10^3^	
	*t*_therm_ (263 K)	s	101^c^	8.6 × 10^5^	
(R1) IOIO + O_3_ → IOIO_4_	ZPE	kcal mol^−1^	−10.8	−1.5	
	*G* (298 K)	kcal mol^−1^	0.5	9.5	
	*k*_1_ (298 K)	molec cm^3^ s^−1^	Collision limit	2.7 × 10^−14^^d^	≥1.1 × 10^−13^^g^
	*t* (40 ppbv O_3_)	s	10^−2^	37	≤10
(R2) IOIO_4_ + H_2_O → HIO_3_ + HOI + ^(1)^O_2_	ZPE	kcal mol^−1^	4.5	5.1	
	*G* (298 K)	kcal mol^−1^	14.6	14.6	
	*k*_2_ (298 K)	molec cm^3^ s^−1^	8.6 × 10^−16^^h^	5.7 × 10^−16^^i^	~2.0 × 10^−16^^k^
	*t* (10% RH)^j^	s	0.015	0.023	~0.063

Bond dissociation energy (BDE) and proton affinity are shown to benchmark the accuracy of theory. The IOIO lifetime against thermal decomposition, *t*_therm_, is predicted to be much longer than previously thought by the theory used in this study. For reactions (R1) and (R2): zero-point corrected energies (ZPE), Gibbs free energies *G*, rate coefficients *k*, typical lifetime *t* against reaction with O_3_ or H_2_O. Experimentally derived reaction rate coefficients are corroborated by theory. IOIO is quantitatively converted into HIO_3_, HOI and H_2_O under typical atmospheric conditions.

^a^CCSD(T)/aug-cc-pVTZ+LANL2DZ//M062X/aug-cc-pVDZ+LANL2DZ, Gomez-Martin et al. ^34^, Kumar et al. ^47^, used in this work for comparison with literature.

^b^CCSD(T)/CBS(T,Q)//M062X/aug-cc-pVTZ-PP.

^c^Saiz-Lopez et al. ^40^ literature review.

^d^TS1 energy changes of 1.3 or 2.6 kcal mol^−1^ correspond to a change in the rate constant of a factor of 10 or 100, respectively.

^e^JPL Publication 19-5 (ref. ^48^).

^f^Ghanty and Gosh^49^.

^g^*k*_1_(263 K) = 1.5 × 10^−13^ molec cm^3^ s^−1^ assuming efficient IOIO wall loss. *k* (298 K) is calculated using the theory-predicted temperature dependence.

^h^MESMER effective rates including the effect of excess energy (Supplementary Section 3.3); thermal rate of 4.7 × 10^−18^ molec cm^3^ s^−1^.

^i^MESMER effective rates including the effect of excess energy (and neglecting the pre-reactive complex; see Supplementary Section 3.3 for details); thermal rate of 8 × 10^−19^ molec cm^3^ s^−1^.

^j^10% RH at *T* = 298 K, equivalent to 8 × 10^16^ molec cm^−3^.

^k^*k*_2_(263 K), based on marginal detection of IOIO_4_; compare Extended Data Fig. 3 and Supplementary Section 2.4.

The transition states in Fig. 3 translate into the rate coefficients for reactions (R1) and (R2) at 298 K as shown in Table 1 (for temperature dependencies see Supplementary Fig. 6). Notably, the experimentally derived *k*_1_ ≥ 1.5 × 10^−13^ molec^−1^ cm^3^ s^−1^ is supported within the error margins of theory and maintains the quantitative conversion of IOIO into HIO_3_ even at low O_3_ concentrations (Supplementary Fig. 1). Our results led to a reassessment of the thermal lifetime of IOIO, which is predicted to be substantially longer than previously thought (Table 1), consistent with observations of IOIO (Extended Data Fig. 3), and its persistently quantitative conversion into HIO_3_ even at extremely low O_3_ concentrations at 263 K (Supplementary Fig. 5 and Supplementary Section 3). Reaction (R2) is predicted to proceed with *k*_2_ = 5.7 × 10^−16^ molec^−1^ cm^3^ s^−1^ at 298 K (Table 1 and Supplementary Fig. 6), corresponding to a typical conversion of IOIO_4_ into HIO_3_ within fractions of a second. Competing pathways of IOIO_4_ into other products than HIO_3_ were investigated (Supplementary Fig. 4, Supplementary Table 4 and Supplementary Section 3), but found to be unlikely. The marginal detection of IOIO_4_ (Extended Data Fig. 3 and Supplementary Section 2.4) is consistent with a value of *k*_2_ ≈ 2.0 × 10^−16^ molec^−1^ cm^3^ s^−1^ at 263 K. The detection of IOIO_4_ at the observed levels suggests that reaction (R2) is enhanced by water reacting with hot IOIO_4_ (Supplementary Section 3.3); assuming a lower *k*_2_ from thermalized IOIO_4_ leads to IOIO_4_ accumulation in the extended model that is not observed. We recommend temperature-dependent rate coefficients for *k*_1_ and *k*_2_ for the development of atmospheric models (Supplementary Section 3). Overall, the theory-predicted rates support the experimentally derived rates within the uncertainty of the calculations.

### Atmospheric observations

The laboratory-derived mechanism can explain field measurements of HIO_3_ concentrations in the remote free troposphere. We use concurrent measurements of HIO_3_ (in situ, NO_3_^−^-CIMS), IO radicals (near-observatory, MAX-DOAS) and particle surface area measurements at the Maïdo observatory^41^ to assess the relevance of CLOUD findings in the real world. The observatory is located in the southern Indian Ocean on Réunion Island at an elevation of 2,200 m, and is frequently exposed to lower free tropospheric air (mornings) and anabatic orographic flows from the ocean (afternoons). The laboratory conditions at CLOUD closely match the conditions at the Maïdo observatory (Supplementary Table 1 and Methods) regarding IO concentrations (single pptv), condensational sink (~10^−3^ s^−1^) and temperature (~283 K).

**Fig. 4:**
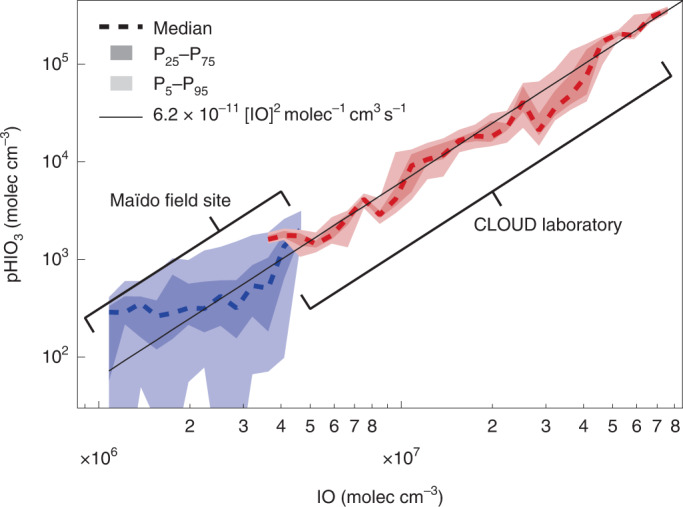
Comparison with field measurements. Good consistency is observed between HIO_3_ production rates measured in the CLOUD laboratory (red) and at the Maïdo field site (blue). IO radical concentrations at CLOUD overlap with those found in the remote lower free troposphere. The solid black line is the IOIO formation rate from IO radicals (at 283 K), and corresponds to the rate-limiting step of HIO_3_ formation under both field and laboratory conditions. Source data

Figure 4 shows pHIO_3_ in the field and laboratory on a common IO radical concentration axis. pHIO_3_ is calculated from HIO_3_ concentrations and the condensation sink surface area, assuming a steady state. IO radical concentrations are measured directly at the Maïdo observatory, and taken from the extended model at CLOUD. The solid line shown in Fig. 4 is not a fit to the data; it corresponds to pIOIO at 283 K and serves as a transfer standard to propagate the mechanistic finding of quantitative IOIO conversion into HIO_3_ from CLOUD (Extended Data Fig. 4) to the field observations. The excellent consistency between the laboratory experiments and field observations demonstrates the atmospheric relevance of the proposed HIO_3_ mechanism.

The ability of our HIO_3_-formation mechanism to predict simultaneous field measurements of HIO_3_ and IO radicals in the remote free troposphere is anything but trivial (Supplementary Fig. 7), and demonstrates the ability to approximate atmospherically relevant experimental conditions at CLOUD. Interestingly, HIO_3_ concentrations at Maïdo increase rapidly already under twilight conditions during sunrise (Supplementary Fig. 7 and Supplementary Section 4). He and colleagues^10^ had predicted the efficient formation of iodine oxoacids under cloudy daylight conditions, and Supplementary Fig. 7 provides field evidence in support of the rapid activation of iodine reservoir species into iodine oxoacids in the absence of ultraviolet irradiation.

### Atmospheric implications

The mechanism provides a source of HIO_3_ that is effective even at low iodine concentrations, and will allow atmospheric models to test HIO_3_ field observations. Such model development will also help guide future laboratory experiments and field observations. The near-linear rate law of pHIO_3_ in pI also enables HIO_3_ formation and subsequent particle formation beyond hotspots at lower iodine concentrations in the background atmosphere^31,42,43^.

**Fig. 5:**
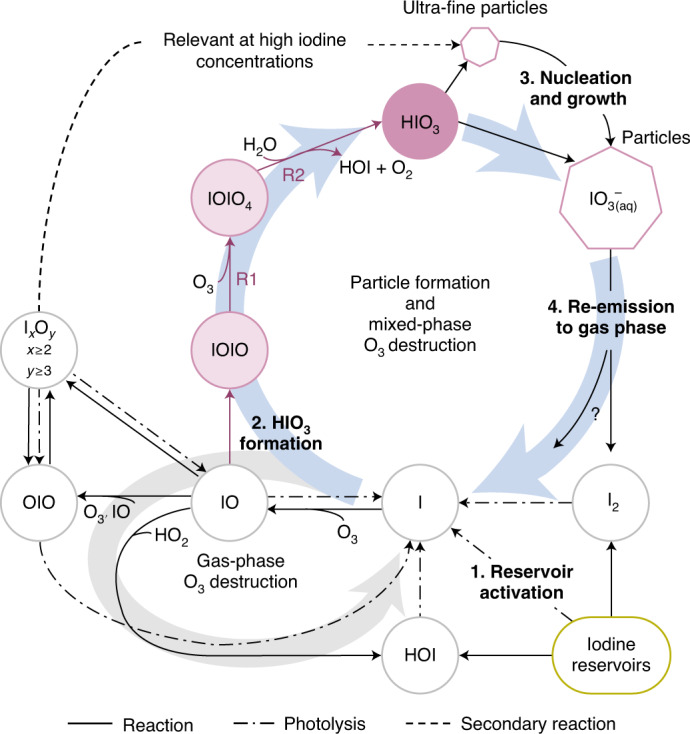
Simplified gas-phase iodine chemistry in the remote atmosphere. After activation of iodine reservoirs (step 1), HIO_3_ is efficiently formed (step 2) and subsequently nucleates and grows particles extremely efficiently (step 3). Iodate (IO_3_^−^) can be reduced and re-emitted to the gas phase (step 4), closing an iodine-catalysed reaction cycle forming particles and destroying O_3_. HIO_3_ formation from IOIO links iodine sources and new particle formation even at lower IO concentrations. This mechanism is currently missing from atmospheric models.

The gas-phase formation mechanism of HIO_3_ we present here facilitates a missing connection between iodine sources and particle formation in atmospheric models, as illustrated in Fig. 5. The activation of iodine reservoir species (Fig. 5, step 1) liberates iodine radicals, which rapidly form IO radicals and HIO_3_ (step 2) via reactions (R1) and (R2). Iodine oxoacid particle formation and growth (step 3) is driven by HIO_3_ in most atmospheric environments. Indeed, recent field observations of particle formation events over the remote Arctic Ocean indicate that all of the observed events were driven by HIO_3_ (ref. ^31^). I_*x*_O_*y*_ species may also contribute locally in coastal hotspots with extremely high iodine concentrations. Freshly nucleated iodine particles are composed almost entirely of HIO_3_ (ref. ^10^); HIO_3_ is a strong acid (p*K*_a_ = 0.8; ref. ^44^) that dissociates to form IO_3_^−^. IO_3_^−^ is known to undergo reduction reactions that ultimately form more volatile iodine species (for example, HOI, I_2_ and IO), which are re-emitted to the gas phase (step 4). Field observations and laboratory experiments show that IO_3_^−^ is reduced via iron redox chemistry, H_2_O_2_, nitrite, photosensitized reactions, photolysis and numerous other species (refs. ^25,26^ and references therein), with the overall effect of recycling iodine to the gas phase. The HIO_3_ formation mechanism thus completes a catalytic iodine reaction cycle, by which a single iodine atom can repeatedly form HIO_3_, driving particle formation. For each HIO_3_ molecule produced from I, three O_3_ molecules are consumed. The re-emission of reduced iodine species thus constitutes a multiphase reaction cycle that destroys O_3_.

That iodine recycling controls the iodine partitioning between the gas and particle phases is corroborated by field measurements of the size-resolved iodine activity in radioactive fallout^45^. Among the primary radioactive elements, ^132^Te, ^137^Cs and ^103^Ru abundances were found to correlate with the aerosol volume distribution, whereas ^131^I correlated with the aerosol surface area distribution instead. These empirical observations hint at efficient recycling occurring on timescales of hours to days, consistent with rapid HIO_3_ formation. Notably, although the reactive uptake of HOI on aerosols is known to be fast^46^, this reaction de facto removes halides from aerosols to the gas phase. A gas-phase source of HIO_3_ adds iodine to particles and, in conjunction with iodine recycling, provides a plausible explanation for the correlation of particulate ^131^I with the aerosol surface area distribution at the molecular level. Particulate IO_3_^−^ is the primary reservoir of total (gas and particle) iodine in the stratosphere^22^. Whether HIO_3_ forms in the stratosphere—and controls iodine partitioning between the gas and particle phases—deserves further study. The HIO_3_-formation mechanism fills a gap in the representation of the geochemical iodine cycle in current atmospheric models.

Iodine particle formation has heretofore been considered to have only limited global importance^19^. This deserves re-evaluation in light of efficient HIO_3_ formation even at low concentrations, the catalytic role of iodine in particle formation, and the increased global iodine source in recent decades. Iodine particle formation is probably already relevant on global scales today, and will become even more important in view of decreasing global sulfur emissions and increasing iodine emissions in a future climate.

## Methods

### CLOUD experiments

Laboratory experiments were carried out at the CERN CLOUD chamber^3,50^ in Geneva, Switzerland as part of the CLOUD12 and CLOUD13 campaigns during 2017 and 2018. The CLOUD chamber is a temperature-controlled, electropolished stainless-steel reaction vessel with a volume of 26.1 m^3^. Experiments were carried out at temperatures of 283 and 263 K. The chamber was operated as a continuous-flow reactor, and ultra-pure N_2_ and O_2_ at 250–300 l min^−1^ were continuously replenished at a pressure of 1 atm, resulting in an air exchange time of ~80 min. Two fans at the top and bottom of the chamber established near-homogeneous mixing (mixing time ~2 min). Trace gases were added at the bottom of the chamber. I_2_ was produced from sublimating iodine crystals (Sigma-Aldrich, 99.999% purity), and concentrations inside the chamber were varied in the range 0.5 pptv < [I_2_] < 330 pptv (typically ~8 pptv). O_3_ was generated from UV irradiation of dry synthetic air, and the chamber was humidified using ultrapurified water, resulting typically in [O_3_] = 40 ppbv (range < 1–80 ppbv) and RH = 40% (<3–90%).

A typical experiment explored the formation of HIO_3_ following the selective photolysis of I_2_ using green light (light-emitting diodes (LEDs) centred at 523 nm, I_2_ photolysis frequencies ${j}_{{{{{\rm{I}}}}}_{2}}\le {6.5}\times {10}^{-3}\,{{{{\rm{s}}}}}^{-1}$) in the presence of O_3_ and humidity (Fig. 1). Actinic frequencies were spectrally determined using a spectrometer and dedicated iodine actinometry experiments (Supplementary Section 6.3). Actinic fluxes of light sources at variable intensity were monitored during actual experiments by photodiodes. Sensitivity studies during individual experiments followed the response in the HIO_3_ concentration to variations in O_3_ (for example, Supplementary Fig. 1), chamber wall loss during variations of the fan mixing speed (for example, Extended Data Fig. 1) and by varying selected environmental parameters. The typical duration of individual experiments varied from a few tens of minutes to a few hours, depending on the experimental conditions.

I_2_ was measured by closed-path CE-DOAS^51^ using the unique ro-vibronic absorption bands between 508 and 554 nm. CE-DOAS is inherently calibrated from knowledge of the absorption cross-section. The I_2_ limit of detection is 8 pptv for an integration time of 10 min. Median I_2_ concentrations were below 8 pptv during most experiments, but elevated to up to 1.7 ppbv to calibrate the Br^−^-MION-CIMS, which also provides precise I_2_ measurements at low concentrations. The Br^−^-MION-CIMS is composed of an atmospheric-pressure interface–time of flight mass spectrometer (APi-TOF) coupled to a chemical ionization unit, using dibromomethane (CH_2_Br_2_) as the reagent gas. The CH_2_Br_2_ is fed into the sheath flow of the inlet and illuminated by a soft X-ray source. The produced bromide anions are directed into the sample flow by a negative electric field, and cluster with neutral molecules (I_2_) in the sample air. The overall uncertainty of the resulting I_2_ time series is estimated to be better than 30%^52^. The I_2_ constraint imposed to the model assimilates the lower bound of the measured I_2_ time series (within the 30% uncertainty), which results in the best closure between measured and predicted HIO_3_. Iodine radical production rates, pI, are calculated from the photolysis rate of I_2_ concentrations.

HIO_3_ was measured by a NO_3_^−^-CIMS system comprising an APi-TOF coupled to a chemical ionization unit that uses nitric acid as the reagent gas. It is used extensively for detecting H_2_SO_4_, highly oxygenated organic molecules and HIO_3_. Details of the instrument used in the present study are provided in ref. ^53^. The NO_3_^−^-CIMS has an ion filter integrated into its sampling line to avoid confusion with ions and charged clusters from the CLOUD chamber. It thus measures only neutral molecules and clusters in CLOUD. The uncertainty of the HIO_3_ measurement is estimated to be 50%.

The characteristic time for the deposition of sticky molecules to the chamber walls is 440 s with standard mixing by the fans (Extended Data Fig. 1), as characterized via H_2_SO_4_ loss rates. The loss to walls is the well-defined dominant sink of HIO_3_. Experiments that formed a large particle surface area (measured by nSEMS, nano-SMPS or long-SMPS) competitive to chamber wall loss were discarded in this study to avoid introducing uncertainty due to the other less-well-defined sinks for HIO_3_ and other iodine species. The HIO_3_ production rates were calculated from measured concentrations under the assumption of a steady state. Periods with rapid changes of HIO_3_ concentration are not considered in, for example, Fig. 2.

### Box modelling

The photochemical box model builds on the framework described in refs. ^22–24^ and represents state-of-the-art iodine chemistry and HO_*x*_ chemistry^25,48^. Here, the model is extended by the chamber auxiliary mechanism, which includes losses of gases to the chamber walls and to particles, losses by dilution and the actinic fluxes of the chamber lights. IO, OIO, IOIO, I_2_O_3_, I_2_O_4_, HI and HIO_3_ are assumed to be lost to the walls with the same rate constant as H_2_SO_4_, the prototypical sticky molecule. Accommodation of molecules to the CLOUD chamber walls is limited by transport, not by diffusion. Thus, the effective wall accommodation coefficient of molecules (most iodine species are reasonably sticky^54–56^, with accommodation coefficients of multiple tens of percent or even unity) used in the model is enhanced over the accommodation coefficient for individual collisions^57^. Extended Data Fig. 1 provides evidence for the efficient loss of iodine species to the chamber walls. The model is constrained by measurements of I_2_, O_3_ and H_2_O, photolysis frequencies (I_2_, IO, OIO, HOI, I_2_O_2_, I_2_O_3_ and I_2_O_4_), temperature and the aforementioned loss mechanisms. HOI is both lost to the walls and produced on the chamber walls through heterogeneous chemistry^14^, which also proceeds in dark conditions. This study did not make an attempt to describe the uptake and release of HOI at the molecular level. An empirical uptake efficiency of 25%, relative to H_2_SO_4_, establishes closure in regard to the temporal evolution and concentrations of HOI (Extended Data Fig. 3). See Supplementary Section 6 for more details.

### Quantum chemical calculations

For the reactants, intermediates, transition states and products in Fig. 3 with multiple possible conformers, a systematic conformer sampling was carried out using the MMFF method in the Spartan ’18 program. The conformer sampling algorithm in Spartan allows for pre-optimization and the elimination of duplicate structures, which is computationally more efficient than other conformer sampling approaches like MS-TOR. Geometry optimization and frequencies were calculated using DFT methods (M062X/aug-cc-pVTZ-PP) with the ultrafine grid, followed by coupled-cluster single-point energy corrections at the CCSD(T)//CBS/aug-cc-pV(T,Q)Z-PP level of theory. Iodine pseudopotentials were taken from the Environmental Molecular Sciences Laboratory (EMSL) basis set library^58,59^. The accuracy of the final energetics is critical to reliably estimate the rate of conversion of IOIO_4_ to HIO_3_, which was simulated using the master equation solver for multi-energy well reactions (MESMER) program.

Final product fractions were calculated using the MESMER program^60^. In the simulation, IOIO + O_3_ was modelled to directly lead to IOIO_4_ using the MesmerILT method with a pre-exponential value of 2.7 × 10^−14^ molec^−1^ cm^3^ s^−1^, which corresponds to the transition-state-theory-derived bimolecular rate. The unimolecular isomerization reactions of intermediate complexes were treated using the SimpleRRKM method with Eckart tunnelling. The MesmerILT method with a pre-exponential value of 2.0 × 10^−10^ molec^−1^ cm^3^ s^−1^ was used for the bimolecular reaction of IOIO_4_ with H_2_O, with the latter set as the excess reactant with a defined initial concentration. All intermediate complexes were assigned as ‘modelled’ with Lennard–Jones potentials of *σ* = 6.5 Å and *ϵ* = 300 K. These are identical to those used by Galvez and colleagues for their iodine systems^61^. MESMER uses the exponential down (Δ*E*_down_) model for simulating the collisional energy transfer; a value of 225 cm^−1^ was used in the simulations, which is within the 175–275 cm^−1^ range recommended by MESMER for nitrogen bath gas.

The energetics of ozonolysis reactions are difficult to calculate accurately using single-reference methods. The inherent uncertainties are probably even more pronounced for complex iodine-containing systems. Although no experimental values are available for the gas-phase ozonolysis reaction of iodine systems, proton affinities (PAs) and BDEs of simple molecules such as HI, HOI, IO and OIO are available. Table 1 shows that the differences between the literature values and the theoretical values calculated in this work are less than 3 kcal mol^−1^ (with the exception of the BDE of OIO). Previous quantum chemical calculations on iodine oxide reactions^34,47^ are included in Table 1 for comparison, highlighting the improved skill of the method used in this study in the coupled-cluster part of the calculation, as benchmarked through comparisons with experimental PAs and BDEs. Previous studies used a double-zeta basis set (LanL2DZ) for I atoms, but a larger triple-zeta basis set (aug-cc-pVTZ) for O and H atoms, leading to substantial overestimation of the exothermicity of bond-forming reactions involving iodine. Our approach uses a large basis set for all atoms, substantially reducing this overestimation.

### Field measurements

The field data were collected during an intensive operating period in April 2018 at the Maïdo observatory^41^, Réunion island, southern Indian Ocean (21° S, 55° E). The observatory is located at 2,200 m above sea level and is frequently exposed to lower free tropospheric air (mornings) and flows from the ocean (afternoons). Near-instrument altitude volume mixing ratios of IO radicals were retrieved from CU MAX-DOAS scattered sunlight observations. The retrieval^62,63^ leverages the high sensitivity of the limb viewing geometry to the atmospheric layers nearest to the instrument altitude, allowing for the parameterization of aerosol effects on the observed light path. Gas-phase HIO_3_ was measured directly by a NO_3_^−^-CIMS system using a methodology similar to that used in the laboratory experiments. The instrument was calibrated in the field in its actual field campaign sampling configuration by in situ-produced H_2_SO_4_, which resulted in a calibration factor of *c* = 1.7 × 10^10^ molec cm^−3^. This same calibration factor was used for all quantifications, so the determined concentrations here represent lower limits. The uncertainty of the determined [HIO_3_] was estimated similarly as [H_2_SO_4_], at −50% and +100% following the work in ref. ^64^. Particles were size-selected by a differential mobility particle sizer and counted with a condensation particle counter to determine the available particle surface area. The box modelling constraints are described in Supplementary Section 4.1. TUV calculated spectral fluxes^65^ were used to determine the photolysis frequencies of individual iodine species.

## Online content

Any methods, additional references, Nature Research reporting summaries, source data, extended data, supplementary information, acknowledgements, peer review information; details of author contributions and competing interests; and statements of data and code availability are available at 10.1038/s41557-022-01067-z.

## Supplementary information

Supplementary Information Supplementary discussion, Figs. 1–11 and Tables 1–11.

## Data Availability

The output files of quantum chemical calculations and a MESMER input file are provided in the public data repository at 10.5281/zenodo.6637910. The box model supporting the findings of this study is described in detail in the Supplementary Information (Supplementary Tables A5–A9 and text). Source data are provided with this paper.

## Source data

Source Data Fig. 1 Plain text data.
Source Data Fig. 2 Plain text data.
Source Data Fig. 4 Plain text data.
Source Data Extended Data Fig. 1 Plain text data.
Source Data Extended Data Fig. 2 Plain text data.
Source Data Extended Data Fig. 3 Plain text data.
Source Data Extended Data Fig. 4 Plain text data.
