# Supplementary material for: The gas-phase formation mechanism of iodic acid as an atmospheric aerosol source
Source: Nat Chem. 2022 Nov 14;15(1):129–35. doi: 10.1038/s41557-022-01067-z (PMC9836935; doi:10.1038/s41557-022-01067-z)
Supplement: Supplementary file 1 — Supplementary discussion, Figs. 1–11 and Tables 1–11. [file 41557_2022_1067_MOESM1_ESM.pdf]

nature chemistry

Article

https://doi.org/10.1038/s41557-022-01067-z

The gas-phase formation mechanism of iodic acid as an atmospheric aerosol source

In the format provided by the authors and unedited

Supplementary Information to: The gas-phase formation mechanism of iodic acid as an atmospheric aerosol source

Henning Finkenzeller[†1,2*], Siddharth Iyer[†3], Xu-Cheng He[4], Mario Simon[5], Theodore K. Koenig[1,2,6], Christopher F. Lee[1,2], Rashid Valiev[7], Victoria Hofbauer[8], Antonio Amorim[9], Rima Baalbaki[4], Andrea Baccarini[10,11], Lisa Beck[4], David M. Bell[10], Lucía Caudillo[5], Dexian Chen[8], Randall Chiu[1,2], Biwu Chu[4,12], Lubna Dada[4,10], Jonathan Duplissy[4,13], Martin Heinritzi[5], Deniz Kemppainen[4], Changhyuk Kim[14,15], Jordan Krechmer[16], Andreas Kürten[5], Alexandr Kvashnin[17], Houssni Lamkaddam[10], Chuan Ping Lee[10], Katrianne Lehtipalo[4,18], Zijun Li[19], Vladimir Makhmutov[17], Hanna E. Manninen[20], Guillaume Marie[5], Ruby Marten[10], Roy L. Mauldin[1,8], Bernhard Mentler[21], Tatjana Müller[5], Tuukka Petäjä[4], Maxim Philippov[17], Ananth Ranjithkumar[22], Birte Rörup[4], Jiali Shen[4], Dominik Stolzenburg[4,23], Christian Tauber[23], Yee Jun Tham[4,24], António Tomé[25], Miguel Vazquez-Pufleau[23], Andrea C. Wagner[1,2,5], Dongyu S. Wang[10], Mingyi Wang[15], Yonghong Wang[4,12], Stefan K. Weber[5,20], Wei Nie[26], Yusheng Wu[4], Mao Xiao[10], Qing Ye[8], Marcel Zauner-Wieczorek[5], Armin Hansel[21], Urs Baltensperger[10], Jérome Brioude[27], Joachim Curtius[5], Neil M. Donahue[8], Imad El Haddad[10], Richard C. Flagan[15], Markku Kulmala[4,26,28], Jasper Kirkby[5,20], Mikko Sipilä[4], Douglas R. Worsnop[4,16], Theo Kurten[7*], Matti Rissanen[3] and Rainer Volkamer[1,2*]

[1]Department of Chemistry, University of Colorado Boulder, Boulder, 80309, CO, USA. [2]Cooperative Institute for Research in Environmental Sciences, University of Colorado Boulder, Boulder, 80309, CO, USA. [3]Aerosol Physics Laboratory, Physics Unit, Faculty of Engineering and Natural Sciences, Tampere University, Tampere, 33720, Finland. [4]Institute for Atmospheric and Earth System Research, University of Helsinki, Helsinki, 00560, Finland. [5]Institute for Atmospheric and Environmental Sciences, Goethe University Frankfurt, Frankfurt, 60438, Germany. [6]now at: State Key Joint Laboratory of Environmental Simulation and Pollution Control, BIC-ESAT and IJRC, College of Environmental Sciences and Engineering, Peking University, Beijing, 100871, China. [7]Department of Chemistry, University of Helsinki, Helsinki, 00014, Finland. [8]Center for Atmospheric Particle Studies, Carnegie Mellon University, Pittsburgh, 15213, PA, USA. [9]CENTRA and Faculdade de Ciências da Universidade de Lisboa, Lisboa, 1749-016, Portugal. [10]Laboratory of Atmospheric Chemistry, Paul Scherrer Institute, 15 avenue René Cassin, Villigen, 5232, Switzerland. [11]Extreme Environments Research Laboratory, École Polytechnique Fédérale de Lausanne, Lausanne, Switzerland. [12]now at: Research Center for Eco-Environmental Sciences, Chinese Academy of Science, Beijing, China. [13]Helsinki Institute of Physics (HIP) / Physics, Faculty of Science, University of Helsinki, Helsinki, 00014, Finland. [14]School of Civil and Environmental Engineering, Pusan National University, Busan, 46241, Republic of Korea. [15]Division of Chemistry and Chemical Engineering, California Institute of Technology, Pasadena, 91125, CA, US. [16]Aerodyne Research, Billerica, 01821, MA, USA. [17]P.N. Lebedev Physical Institute of the Russian Academy of Sciences and Moscow Institute of Physics and Technology (National Research University), Moscow, Russia. [18]Finnish Meteorological Institute, Helsinki, 00560, Finland. [19]Department of Applied Physics, University of Eastern Finland, Kuopio, 70200, Finland. [20]CERN, the European Organization for Nuclear Research, CH-1211 Geneva 23, Switzerland. [21]Institute of Ion and Applied Physics, University of Innsbruck, Technikerstrasse 25, Innsbruck, 6020, Austria. [22]School of Earth and Environment, University of Leeds, LS2 9JT, Leeds, United Kingdom. [23]Faculty of Physics, University of Vienna, Vienna, 1090, Austria. [24]School of Marine Sciences, Sun Yat-sen University, Zhuhai, 519082, China. [25]IDL-Universidade da Beira Interior, Covilhã, 6201-001, Portugal. [26]Joint International Research Laboratory of Atmospheric and Earth System Research, School of Atmospheric Sciences, Nanjing University, Nanjing, China. [27]LACy UMR8105, Université de la Réunion, Saint Denis, 97400, Reunion. [28]Aerosol and Haze Laboratory, Beijing Advanced Innovation Center for Soft Matter Sciences and Engineering, Beijing University of Chemical Technology (BUCT), Beijing, China.

† These authors contributed equally to this work.
*Corresponding authors:
henning.finkenzeller@colorado.edu; theo.kurten@helsinki.fi; rainer.volkamer@colorado.edu

Contents

1 Comparison of environmental conditions 4

2 CLOUD laboratory experiments 5
2.1 Evaluation of HIO_3 precursors . . . 5
2.2 Sensitivity studies that vary environmental conditions . . . 6
2.3 Robustness of gas-phase HIO_3 measurements . . . 7
2.4 Measurements and calibrations of other iodine species . . . 8

3 Quantum Chemical Calculations 13
3.1 Additional investigations on the fate of $IOIO_4$ . . . 13
3.2 Sensitivity studies . . . 14
3.3 Guidance for model development . . . 15

4 Maïdo field measurements 18
4.1 Chemical box modelling . . . 19
4.2 Modelled HIO_3 time series . . . 19
4.3 Modelled $I_{y,\mathrm{gas}}$ partitioning . . . 19

5 HIO_3 formation in flow-tube experiments 21
5.1 Sensitivity of HIO_3 formation to humidity . . . 21
5.2 Competition of HIO_3 & I_xO_y in flow tube experiments . . . 21

6 Description of the chemical box-model 23
6.1 Gas-phase reactions . . . 23
6.2 Thermal decomposition reactions . . . 26
6.3 Photochemistry . . . 26

List of figures

1 Sensitivity of HIO_3 to changes in O_3 concentrations under the assumption of different hypothesised mechanisms, and comparison with observations at the CLOUD chamber . . 7
2 HIO_3 production rate as function of I atom production rate, assuming I_xO_y fragmentation 9
3 Fragmentation enthalpies of reagent-ion–analyte adducts . . . 12
4 Reaction coordinate of alternative pathways . . . 13
5 Sinks of IOIO during variations of O_3 . . . 16
6 Temperature dependent rate expressions for reactions R1 and R2 . . . 17
7 Detection of iodine species at the Maïdo field site . . . 18
8 Iodine partitioning at the Maïdo field site . . . 20
9 Formation of HIO_3 at variable humidity in flow tube . . . 21
10 Iodine photochemistry at the CLOUD chamber . . . 27
11 Actinometry experiment to determine photolysis frequency of I_2 due to LS4. . . . 27

List of tables

1 Comparison of iodine environmental conditions at the CERN CLOUD chamber with those in the atmosphere, and previous flow-tube experiments . . . 4
2 Compatibility of different HIO_3 formation mechanisms with laboratory observations . . . 5
3 Calibration factors of the NO_3^--CIMS and Br^--MION-CIMS for detection of iodine species 10
4 Predicted energies and rates for reactions R3 and R4 using theory as in this study and as in the literature. . . . 13
5 Spin-orbit coupling energies of the I_2O_2 + O_3 reaction stationary points. . . . 14
6 Relative spin-orbit coupling energies. . . . 15
7 Gas-phase iodine reactions used in model base case. . . . 23
8 HO_x reactions in photochemical box model . . . 24
9 Gas-phase NOx reactions used in model . . . 25
10 Thermal decomposition rate expressions and lifetimes of iodine species in the box model. 26
11 Photolysis reactions and rates in chemical box model . . . 28

1 Comparison of environmental conditions

Supplementary Table 1: Comparison of iodine environmental conditions at the CERN CLOUD chamber with those in the atmosphere, and previous flow-tube experiments. Adapted and expanded from [1].

Location	I_2 pptv	pI 10^6 molec cm^{-3} s^{-1}	IO pptv	t(IO+IO)[a] s	$pHIO_3$ 10^5 molec cm^{-3} s^{-1}	pI_xO_y[b] 10^5 molec cm^{-3} s^{-1}	$\frac{pI_xO_y}{pHIO_3}$
Mace Head							
day, low tide	20+	100+	4–10+	40–100	6–40		
day, high tide	5	30	2–7	60–200	2–20		
night	few 10	0.5	0.5–4	100–800	0.1–6		
Open ocean	1	6	0.5–1	$10^3 - 10^4$	0.1–0.4		
Maïdo	0.5	0.5	0.15	3300	0.006	$5 \cdot 10^{-4}$	0.01
CLOUD							
median	8	0.1	0.8	500	0.2	0.09	0.4
min–max	0.5–330	0.02–1.4	0.2–3	130–2000	0.01–4	0.002–2	0.1–0.6
Flow tube[c]			10^4	0.04	$4 \cdot 10^6$	$2 \cdot 10^9$	400

[a]lifetime of IO against self-reaction (oligomerisation)
[b]formation rate of iodine oxide clusters, approximated by sum of I_2O_3, and I_2O_4 formation
[c]conditions as in [2], Fig. 4; using $[O_3] = 1.5 \cdot 10^{15}$ molec cm^{-3}

Supplementary Table 1 shows a comparison of iodine chemical conditions in different laboratory and atmospheric environments. IO radical volume mixing ratios (VMR) in the atmosphere vary from fractions of pptv in the free troposphere (Maïdo) [3–5] and over the open ocean [5–7] to several pptv in coastal hot spots (Mace Head) [8, 9]. IO radical concentrations at CLOUD compare to or approach these atmospheric conditions. In flow tube experiments with lower residence times, precursor concentrations (i.e., IO) generally need to be elevated above atmospheric levels to accelerate their chemical conversion. $t(IO + IO)$, the lifetime of IO against self-reaction, is shown here as proxy for the typical time between collisions of iodine species. Depending on the specific experimental setup employed, precursor concentrations might differ by many orders of magnitude from those in the atmosphere.

Supplementary Table 1 further illustrates the shift in the chemical regime towards oligomerisation reactions as a consequence of elevated precursor concentrations. The production rate $pHIO_3$ is estimated here as the formation rate of I_2O_2 from the self reaction of IO (compare Fig. 4 and Extended Data Fig. 4).The formation rate of larger I_xO_y species is estimated as the sum of the I_2O_3 and I_2O_4 formation rates. Numbers are only given for the Maïdo field site, CLOUD, and a flowtube approximating conditions as in [2] as the estimation of the latter requires an estimate of OIO concentrations. Finally, the ratio $pI_xO_y/pHIO_3$ indicates the branching between the formation of large I_xO_y and HIO_3. A ratio larger than one indicates preference towards polymerisation reactions over HIO_3 formation. This simplified approach clearly shows that HIO_3 is favoured under most atmospheric conditions, but direct pathways to HIO_3 are in competition with, and increasingly suppressed by polymerisation reactions at progressively higher precursor concentrations. The extrapolation of experimental findings under conditions orders of magnitude away from atmospherically relevant conditions is inherently difficult. CLOUD is unique in that it allows to conduct controlled soft experiments that reduce the need for extrapolation.

2 CLOUD laboratory experiments

2.1 Evaluation of HIO_3 precursors

Supplementary Table 2: Compatibility of different HIO_3 formation mechanisms with laboratory observations in regard to variations in O_3 and H_2O, mass closure, rise and decay time, and variation of wall-loss time (fan speed). Pluses indicate compatibility, circles marginal compatibility, and minuses incompatibility, respectively. The formation from IOIO is the only mechanism compatible with all observations. See text for details.

	mechanism	parameter					
		O_3	H_2O	mass closure	appearance time	decay upon lights off [c]	k_{wall} [d]
1	$OIO + OH \rightarrow HIO_3$			none[a]			
2	$I \cdot H_2O + O_3 \rightarrow HIO_3 + OH$	∘	−	∘	−	+	−
3	$IO \cdot H_2O + O_3 \rightarrow HIO_3 + HO_2$	−	−	−	−	+	−
4	$I_2O_3 + H_2O \rightarrow HIO_3 + HOI$	+	−	+	∘	∘	∘
5	$I_2O_4 + H_2O \rightarrow HIO_3 + HIO_2$	+	−	−	−	−	∘
6	$OIO + O_3 \rightarrow IO_3 + O_2$,<br>$IO_3 + H_2O \rightarrow HIO_3 + OH$	−	∘	+	∘	+	+
7[b]	$IOIO + O_3 \rightarrow IOIO_4$,<br>$IOIO_4 + H_2O \rightarrow HIO_3 + HOI + O_2$	+	+	+	+	+	+
8[b]	$IOIO + O_3 \rightarrow IO_3 + I + {}^{(3)}O_2$,<br>$IO_3 + H_2O \rightarrow HIO_3 + OH$	+	+	+	+	+	+

[a] not major pathway, and not a HIO_3 source in HO_x-free (UV-dark) conditions
[b] both pathways lead to HIO_3 and HOI, and are not distinguished experimentally at CLOUD
[c] reproduces temporal response of HIO_3 to turning lights off
[d] reproduces sensitivity of HIO_3 towards fan-speed variations, see Extended Data Fig. 1

We conducted box-modelling sensitivity studies to evaluate the feasibility of a variety of HIO_3 precursors regarding response to O_3 and humidity variations, mass closure, timing, and losses to the chamber walls. The effective rate constants of the considered reactions were varied during the sensitivity studies to improve mass closure for specific conditions. The results shown in Extended Data Fig. 2 and Supplementary Table 2 reveal unique insights about precursors, and pathways to form HIO_3:

1. OIO + OH: While the reaction appears feasible [10], it does not produce HIO_3 in absence of HO_x radicals under green-light-only conditions. Even in UV-bright conditions including HO_x, it could not explain the observations of HIO_3, as OH is rapidly lost to species more abundant than OIO.
2. $I \cdot H_2O + O_3 \rightarrow HIO_3 + OH$: This source would be sensitive to humidity if the conversion of I radicals is not quantitative. Additionally, the production of HIO_3 would start immediately after the light onset, which is not observed (Extended Data Fig. 2). The superlinear response to stirring requires a reasonably long-lived intermediate, but the water adducts are expected to form instantaneously. However, Extended Data Fig. 2, and Supplementary Table 2 assume a rate constant near the kinetic limit, which would be needed to reach anything near mass closure. We conclude that this reaction cannot explain the observations of HIO_3.

3. $IO \cdot H_2O + O_3 \rightarrow HIO_3 + HO_2$: The formation of IO from $I + O_3$ is very fast, such that the same rationale applies as for $I \cdot H_2O + O_3$: The formation of HIO_3 would start immediately and depend on humidity, which is not observed.
4. $I_2O_3 + H_2O \rightarrow HIO_3 + HOI$: This source is robust against variations in O_3, based on the efficient conversion of I into IO. The model predicts appreciable amounts of I_2O_3 to form, but for a non-quantitative I_2O_3 conversion a sensitivity of HIO_3 formation to humidity would result. Also, I_2O_3 forms too slowly to qualify as a major source for HIO_3.
5. $I_2O_4 + H_2O \rightarrow HIO_3 + HIO_2$: I_2O_4 forms even later than I_2O_3, incompatible with the empirical rapid formation of HIO_3. The presence of I_2O_4 in measurements is incompatible with a non-quantitative conversion by H_2O.
6. $OIO + O_3 \rightarrow IO_3 + {}^{(3)}O_2$, $IO_3 + H_2O \rightarrow HIO_3 + OH$: OIO forms sufficiently fast from the self-reaction of IO, but OIO does not get quantitatively converted into IO_3 radicals. A sensitivity of HIO_3 formation to O_3 would be expected, in contrast to the experimental findings. The mechanism could be robust against variations in humidity, as long as the $IO_3 + H_2O$ conversion is quantitative even at low humidity.
7. The proposed mechanism (R1) and (R2) is compatible with all laboratory observations. It reproduces the observed delay in HIO_3 formation well, and predicts the observed HIO_3 concentrations well at 283 K and 263 K, high and low HIO_3 concentrations (Extended Data Fig. 2 and Fig. 2), high and low O_3 and humidity (Supplementary Fig. 1 and Extended Data Fig. 4). IOIO is unique among precursors for HIO_3 in this respect.
8. $IOIO + O_3 \rightarrow IO_3 + I + {}^{(3)}O_2$, $IO_3 + H_2O \rightarrow HIO_3 + OH$: IOIO could potentially form HIO_3 and OH via IO_3 radical intermediates. Because the mechanism would effectively produce HIO_3 and HOI similar as the proposed mechanism ($OH + I_2 \rightarrow HOI + I$, and $HO_2 + IO \rightarrow HOI + O_2$), it can not be ruled out by the experimental constraints in this study. However, it is not corroborated by quantum chemical calculations (Supplementary Section 3).

The comprehensive and unique compatibility of IOIO as precursor shown in Extended Data Fig. 2is further corroborated by sensitivity studies shown in Extended Data Fig. 4, Supplementary Figs. 1, 5, Extended Data Fig. 1as summarised in Supplementary Table 2, and provides strong experimental and box-modelling evidence in support of the proposed mechanism. The corroborating evidence from sensitivity studies that varied environmental conditions is discussed in Supplementary Section 2.2.

2.2 Sensitivity studies that vary environmental conditions

Experiments at the CLOUD laboratory varied the physical and chemical environment to elucidate the HIO_3 formation mechanism. Parameters varied include variation in the pI (Fig. 2), $[O_3]$ (Extended Data Fig. 4, Supplementary Figs. 1, 5), $[H_2O]$, T (Extended Data Fig. 4).

Extended Data Figure 4 shows an $[O_3]$ sensitivity study at 263 K which varied $[O_3]$ over a range of approximately 2 orders of magnitude (<0.6–40 ppbv) by stopping the injection of O_3 and diluting it out of the chamber over the course of approximately 4 h (Supplementary Fig. 5). Lights stayed on during the experiment, HIO_3 was continuously produced. The concentrations of HIO_3 did not vary by 2 orders of magnitude, as expected if HIO_3 production was first order in $[O_3]$ (dashed line in Extended Data Fig. 4A), but remained constant within the variability of the measurements. The mechanism in the extended model reproduces this observation, as IOIO is predominately converted into $IOIO_4$ even at the lowest $[O_3]$ (Supplementary Fig. 5). Mechanisms in which O_3 is a rate limiting reagent (for the conditions probed) are difficult to reconcile with the observed insensitivity to O_3 (Supplementary Fig. 1).

Humidity was varied at 283 K, by a factor 30 (3–90 % relative humidity). Similar as for O_3, a first order rate dependency is absent. Mechanisms in which H_2O is a rate limiting reagent (for the conditions probed) lead to an expectation for variability that is indicated by the dashed line in Extended Data Fig. 4B; such variability is not observed, and such mechanisms are thus difficult to reconcile with the observed insensitivity to H_2O.

Extended Data Figure 4 shows the effect of temperature on the formation of HIO_3. Data at 263 K and 283 K do not indicate a pronounced temperature sensitivity at these moderate temperatures, and the extended model predicts complete conversion of IOIO into HIO_3 for both temperatures.

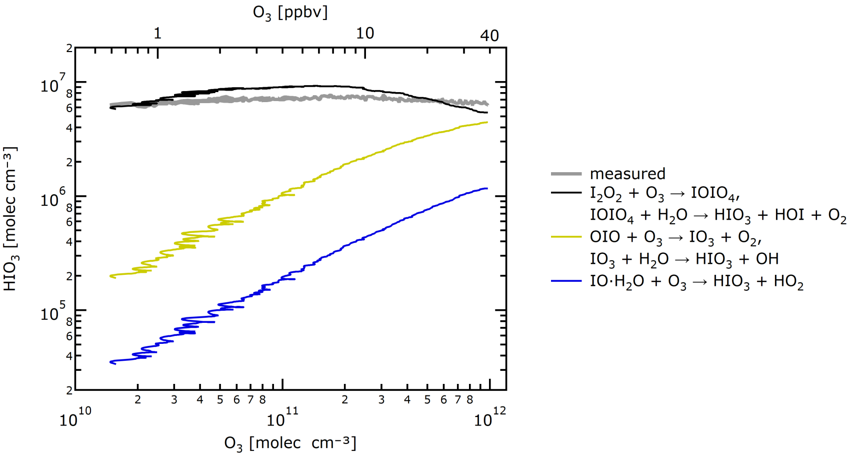

Supplementary Fig. 1: Sensitivity of HIO_3 to changes in O_3 concentrations under the assumption of different hypothesised mechanisms, and comparison with observations at the CLOUD chamber.

2.3 Robustness of gas-phase HIO_3 measurements

HIO_3 was measured by multiple instruments: NO_3^--CIMS, Br^--MION-CIMS (Extended Data Fig. 3) [11–13], NO_3^--HOxROx-CIMS [14] (not shown), and water cluster CIMS (H_3O^+-CIMS) [15], not shown).

The NO_3^--CIMS was chosen as reference instrument for the determination of HIO_3 concentrations, consistent with previous studies [1, 13, 16]. For the NO_3^--CIMS, most signal ($\sim$ 90 %) attributed to HIO_3 is $HIO_3NO_3^-$ and $HIO_3HNO_3NO_3^-$. Only a small fraction ($\sim$ 10%) of HIO_3 dissociates to form IO_3^-, which is not lost but counted towards HIO_3. The time series of IO_3^- correlates near-perfectly with the $HIO_3NO_3^-$ and $HIO_3HNO_3NO_3^-$ time series, corroborating the origin from the same molecule, HIO_3. The reported HIO_3 concentrations in this study are provided by NO_3^--CIMS, as this measurement was robust in all reported cases, inter-compared and validated by other mass spectrometers.

When working in the baseline mode, the NO_3^--HOxROx-CIMS is essentially the same as the NO_3^--CIMS. HIO_3 time traces from independently calibrated NO_3^--CIMS and NO_3^--HOxROx-CIMS were compared for selected periods and the difference was well within the reported HIO_3 measurement uncertainty of [−33%/+50%]. H_3O^+-CIMS and Br^--MION-CIMS continuously traced HIO_3 concentrations in the chamber. However, rigorous calibrations for H_2SO_4 and HIO_3 were not carried out for these two instruments, and the data only provide qualitative corroboration.

Fragmentation of larger I_xO_y cannot explain the observed HIO_3

Recent flow tube laboratory studies have suggested that measurement signals attributed to HIO_3 may be measurement artefacts arising from re-arrangement or fragmentation of larger I_xO_y upon ionisation and detection using NO_3^--CIMS [2, 17]. Under these extreme iodine concentrations polymerisation reactions dominate, and large I_xO_y may indeed contribute some HIO_3. However, at the probed atmospherically relevant conditions (Supplementary Table 1) we find that the observed concentrations of HIO_3 cannot be explained as measurement artefacts of fragmenting I_xO_y species for the following reasons:

1. timing: HIO_3 is measured rapidly after turning on lights (Fig. 1), before larger I_xO_y start to form; I_xO_y larger than I_2O_2 form too slowly to explain the fast appearance time of HIO_3 (Extended Data Fig. 2).

2. rate law: Figure 2 shows an essentially constant HIO_3 yield, if wall losses are accounted for. If large I_xO_y were the source of HIO_3, a pronounced sensitivity of the HIO_3 yield to pI would be expected (Supplementary Fig. 2). This is not the case.
3. mass balance: I_xO_y concentrations are not sufficient to explain HIO_3 concentrations under the probed conditions. This is particularly obvious in the field (Supplementary Fig. 8), where low concentrations and photolysis of I_xO_y [18] lead to concentrations $[I_xO_y] \ll [HIO_3]$. Even if all I_xO_y was fragmenting and detected as HIO_3, the measured concentrations of HIO_3 would be essentially unexplained.
4. Three independent instruments (two nitrate-CIMS and one bromide-CIMS) show good agreement for the measured HIO_3 time series despite using different chemical ionisation schemes. It is difficult to reconcile the results with the detection of I_xO_y as HIO_3, i.e., measurement artefacts would essentially need to show independent of the ionisation scheme and softness used. The $HIO_3 \cdot Br^-$ anion is particularly unlikely to originate from iodine compounds other than HIO_3, and is increasingly being accepted in the literature as a genuine HIO_3 tracer [17].
5. The detection of IOIO and I_2O_4 at the expected levels, and of I_2O_3 under extreme conditions, corroborates the ability to detect I_xO_y species quantitatively, without any apparent fragmentation of I_xO_y species limiting our analysis.
6. More specifically, I_2O_3 fragmentation in NO_3^--CIMS was suggested by Gomez-Martin et al., 2022 [17]. We estimate the MESMER derived overall rate coefficients at 298 K, 1 atm for the two competing reactions:
   R5: $I_2O_3 + HNO_3NO_3^- \rightarrow IONO_2 + HNO_3IO_3^-$
   R6: $I_2O_3 + HNO_3NO_3^- \rightarrow I_2O_3NO_3^- + HNO_3$
   The overall bi-molecular rate coefficients for $k_5 = 5.6 \cdot 10^{-12}\,\mathrm{cm^3\,molec^{-1}\,s^{-1}}$, and $k_6 = 1.5 \cdot 10^{-9}\,\mathrm{cm^3\,molec^{-1}\,s^{-1}}$. These rate coefficients assume the initial $I_2O_3 + HNO_3NO_3^-$ collision rate coefficient (pre-exponential factor) of $1.5 \cdot 10^{-9}\,\mathrm{cm^3\,molec^{-1}\,s^{-1}}$, which is a reasonable neutral–ion collision rate coefficient. The yield towards I_2O_3 detection is thus close to unity, and fragmentation pathways are essentially negligible (< 0.003), i.e., too slow to contribute significant IO_3^- signal over the few tens to hundreds ms residence time inside the ion molecule reaction chamber of the NO_3^--CIMS.
7. $IOIO_4$ formation is shown on the computational reaction coordinate to be a kinetically and thermodynamically plausible HIO_3 precursor (favourable product of IOIO + O_3). Furthermore, $IOIO_4$ is detected in concentrations consistent with the proposed HIO_3 formation mechanism.
8. Finally, at CLOUD we have previously shown with an atmospheric pressure interface time-of-flight mass spectrometer (i.e., not using chemical ionisation) the formation of aerosol particles by the sequential addition of HIO_3 [13]. Critically, the measured collision rates between neutral HIO_3 monomers and charged clusters containing up to 11 iodine atoms match exactly the theoretical expectations, where the enhancement factor for charged versus neutral collision rate coefficients is determined by the intrinsic molecular properties of HIO_3 [1, 13]. This corroborates that a) gas phase HIO_3 is measured beyond analytical doubt, b) concentrations are well calibrated, and c) ion-induced nucleation from iodine is driven by HIO_3 at the measured concentrations.

2.4 Measurements and calibrations of other iodine species

Extended Data Figure 3 shows time series of iodine species during CLOUD 13, at $T = 263\,\mathrm{K}$, which span a range of $\sim 10^6$–$10^8\,\mathrm{molec\,cm^{-3}}$. The concentration predictions by the model base case (blue), and extended model (red) are complemented by the measured time series of the NO_3^--CIMS and Br^--MION-CIMS (right axes show normalised counts per second, ncps). The scaling between the left and right axes reflect the calibration factors shown in Supplementary Table 3. To estimate the CIMS sensitivities towards detection of iodine species, we explored cluster fragmentation energies into various products (Supplementary Fig. 3).

The base case and extended model predict very similar IO concentrations. IO radical sources and sinks are largely independent of the added reactions in the extended model, and the IO radical formation and sink kinetics are well described by theory. This is especially true soon after the start of illumination,

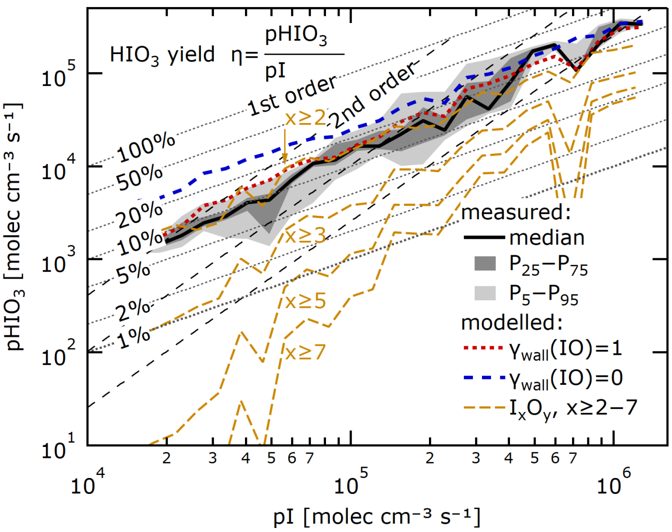

Supplementary Fig. 2: HIO_3 yield η, and rate order (amendment of Fig. 2). The HIO_3 production rate $pHIO_3$ scales in first order with the I atom production rate pI (median, solid line, and 25–75% and 5–95% inter-percentile ranges, dark and light grey shading). The pronounced pI sensitivity of I_xO_y as hypothetical HIO_3 precursors (yellow lines) is incompatible with measurements. I_xO_y concentrations are estimated as upper limit in an amended version of the base case model that contains a tentative mechanism of I_xO_y, $x \geq 3$ formation [2].

when IO sources other than the recombination of I radicals with O_3 are absent (Fig. 1). The associated uncertainty in [IO] is therefore determined by the uncertainty in the measurement of $[I_2]$ (30%) and its photolysis rate (15%), approximately 35%. The high degree of certainty in [IO] predictions justifies the calibration of the time series measured by the Br^--MION-CIMS. This approach to calibrate CIMS is essentially equivalent to kinetic approaches to calibrate the IO radical absorption cross section in molecular spectroscopy, which is known to within few percent [19, 20]. We find that IO is detected with approximately 50 % of the collision (maximum) efficiency by Br^--MION-CIMS, compared to the detection at the kinetic limit for I_2, I_2O_4, HIO_3, and H_2SO_4. This is compatible with only a moderate cluster stability (Supplementary Table 3). Evidence from instrument characterisation experiments (voltage scanning) corroborates that $IO \cdot Br^-$ de-clusters within the instrument for the tuning parameters used during the campaign. This explanation is further corroborated by a slightly decreased sensitivity at 283 K, in line with enhanced de-clustering under warmer conditions.

OIO concentration predictions differ by approximately a factor of 2 between the model base case and the extended model. The reason for reduced OIO concentrations in the extended model is the higher thermal stability of IOIO, which de-facto removes a source of OIO and I. For the Br^--MION-CIMS, assuming the same sensitivity for OIO as for IO (similar cluster stability, Supplementary Table 3) brings the measured time series into agreement with the extended model predictions. For the NO_3^--CIMS, the comparably low cluster stability suggests a moderate detection efficiency, and empirically a reasonable detection efficiency of ∼15 % is determined.

IOIO is detected spuriously by NO_3^--CIMS, and a ∼10 % detection efficiency is required to establish closure to the concentrations predicted by the model. The fragmentation energy of $I_2O_2 \cdot NO_3^-$ is predicted to be 25.0 kcal mol^{-1} (Supplementary Fig. 3), such that a reasonably efficient detection would be expected. The seemingly low detection efficiency might be an indication for k_1 to be higher than currently used in the extended model, i.e., IOIO could react with O_3 even faster than estimated and required (Supplementary Fig. 5). Under the experimental conditions probed, k_1 is derived as a lower limit, and no firm conclusions on the value of k_1 can be derived.

Supplementary Table 3: Calibration factors c_{cal} and relative calibration factors c_{cal}^{rel} (compared to maximum sensitivity) of the NO_3^--CIMS and Br^--MION-CIMS for detection of iodine species at $T = 263\,K$ during conditions as in Fig. 3. Cluster fragmentation enthalpies $\Delta H_{298.15\,K}$ are given as indicator of the stability of the formed ion clusters.

molecule	NO_3^--CIMS			Br^--MION-CIMS		
	$\Delta H_{298.15\,K}$ kcal mol^{-1}	c_{cal} molec cm^{-3} ncps^{-1}	c_{cal}^{rel}	$\Delta H_{298.15\,K}$ kcal mol^{-1}	c_{cal} molec cm^{-3} ncps^{-1}	c_{cal}^{rel}
I_2	26.0 [a]			33.7 [b]	$3.0 \cdot 10^{10}$	100%
IO	23.6 [a]			24.5 [b]	$6 \cdot 10^{10}$	50%
OIO	27.6 [a]	$6 \cdot 10^{10}$	15 %	23.2 [b]	$6 \cdot 10^{10}$	50%
IOIO	34.9 [a]	$1 \cdot 10^{11}$	10 %	43.5 [a]		
$IOIO_4$	35.6 [ae]	$1.04 \cdot 10^{10}$	100 %			
I_2O_3	37.6 [af]	$1.04 \cdot 10^{10}$ [c]	100 %[c]	49.9 [a]		
I_2O_4	45.6 [a]	$1.04 \cdot 10^{10}$	100 %	42.6 [b]	$3.0 \cdot 10^{10}$	100%
I_2O_5	47.6 [d]	$1.04 \cdot 10^{10}$	100 %	53.2 [b]	$3.0 \cdot 10^{10}$ [c]	100 %[c]
HOI	22.8 [a]			29.2 [b]	$1 \cdot 10^{11}$	30%
HIO_3	38.5 [a]	$1.04 \cdot 10^{10}$	100 %	35.2 [gd]	$3.0 \cdot 10^{10}$	100%
$IONO_2$	41.6 [a]			50.1 [a]		

[a]this study, using theory at level CCSD(T)/aug-cc-pVTZ-PP//M062X/aug-cc-pVTZ-PP
[b][12], using theory at level DLPNO-CCSD(T)/def2-QZVPP//wB97X-D/aug-cc-pVTZ-PP
[c]predicted sensitivity based on cluster fragmentation enthalpy
[d]this study, using theory at level DLPNO-CCSD(T)/def2-QZVPP//wB97X-D/aug-cc-pVTZ-PP, as in [12]
[e]for fragmentation to $OIONO_2 + IO_4^-$, $\Delta H(IOIO_4 \cdot NO_3^- \rightarrow IOIO_4 + NO_3^-) = 39.9\,kcal\,mol^{-1}$
[f]for fragmentation to $IONO_2 + IO_3^-$, $\Delta H(I_2O_3 \cdot NO_3^- \rightarrow I_2O_3 + NO_3^-) = 40.2\,kcal\,mol^{-1}$
[g]$\Delta H(HIO_3 \cdot Br^- \rightarrow IO_3^- + HBr) = 29.9\,kcal\,mol^{-1}$, but product IO_3^- is detected and accounted for

I_2O_3 should be detectable by both the NO_3^--CIMS (Supplementary Fig. 3) and Br^--MION-CIMS with reasonable efficiency, based on cluster fragmentation enthalpies, but it is generally absent from measurements in both instruments. We hypothesise that the model is incomplete, and additional sink mechanisms for I_2O_3 might be relevant. Specifically, the reaction $I_2O_3 + O_3 \rightarrow I_2O_4 + O_2$ has been discussed previously in the literature [2, 21]. While there is significant uncertainty in the predicted rate coefficients ($k = 8 \cdot 10^{-14}$ [2, manually fitted], $k = 5 \cdot 10^{-16}$ [21, assumed to be equal to $k(IO + O_3)$]), the difference between measurements and model suggests the sink mechanisms to be fast, relative to losses to the chamber wall. Lower concentrations of I_2O_3 relative to the base-case are predicted in the extended model because of lower OIO concentrations, but Extended Data Figure 3 suggests that I_2O_3 is still considerably over-predicted.

I_2O_4 concentrations are expected to be detected efficiently by both the NO_3^--CIMS and Br^--MION-CIMS, based on the cluster formation energy. The extended model reproduces the concentrations measured under the assumption of efficient detection. I_2O_4 is only formed from the OIO self-reaction, and at 263 K its primary sink is loss to the chamber walls. As a consequence of the good prediction of I_2O_4 concentrations by the extended model, it is likely that the OIO concentrations predicted in the extended model are also approximately correct. In the model base case, OIO concentrations are twice as high, and result in I_2O_4 concentrations that are four times higher than in the extended model, which is difficult to reconcile with the measurements.

I_2O_5 is detected spuriously by the NO_3^--CIMS (Extended Data Fig. 3), and could be interpreted as intermediate $IOIO_4$ formed in the extended model. The extended model does not form any I_2O_5, consistent with the lack of gas-phase reactions forming I_2O_5 in the literature. Measured and predicted $IOIO_4$

concentrations generally agree, albeit close to the detection limit. This is taken as evidence in support of the experimentally derived reaction rate constant k_2 (Table 1). A previous laboratory study [17] observed I_2O_5 concentrations to depend inversely on humidity, which we interpret as an additional piece of evidence for the mechanism proceeding via intermediate $IOIO_4$. Interestingly, both $IOIO_4$ and I_2O_5 are detected sensitively by NO_3^--CIMS (Supplementary Fig. 3) and have similar calibration factors (Supplementary Table 3). $IOIO_4$ and I_2O_5 are different molecules with identical mass, but likely exhibit a different hydrolysis behaviour. Under very dry conditions, we observe signals do increase, consistent with the expectations for higher $IOIO_4$ concentrations under less efficient sinks via R2 from the mechanism. However, insufficient control under these extremely dry conditions (i.e., uncertain water vapour concentration, condensation sink, etc.) currently prevents the determination of k_2 from these experiments. In principle, dedicated experiments that measure $IOIO_4/I_2O_5$ with better signal-to-noise, and vary humidity and temperature with good control over the experimental conditions, hold potential to refine temperature dependent estimates of k_2.

HOI is only formed in the extended model, not in the model base case. The sink for HOI is not very well-defined, as HOI is both lost to [22] and produced on the chamber surfaces [23]. HOI is also produced in dark conditions, which explains the baseline between illuminated stages. The properties of the chamber walls (loading, pH, etc.) likely also change during the different experiments. This study did not attempt to represent dark heterogeneous chemistry, but used a constant effective wall uptake coefficient of 25 % (resulting in a typical wall uptake time comparable to the chamber dilution time), which reproduces the establishment time of the steady state and typical decay rates. Under these conditions a detection efficiency of ∼30 % is required to reach closure between the measurements by the Br^--MION-CIMS and the predictions by the extended model. The moderate detection efficiency is supported by the moderate cluster stability and the associated partial fragmentation in the instrument [12].

Previous studies using NO_3^--CIMS found ions with a mass signature of $IONO_2$ [17, 24]. Signals with a $IONO_2$ signature are also detected by NO_3^--CIMS in the NO_x-free laboratory experiments of this study, where $IONO_2$ is not expected to form. Here, the presence of $IONO_2$ signals can be rationalised as multiple other iodine oxides potentially form $IONO_2$ upon ionisation with NO_3^- (Supplementary Fig. 3): $IOIO + NO_3^- \rightarrow IONO_2 + OIO^-$, $IOIO_4 + NO_3^- \rightarrow IONO_2 + OIO_4^-$, and $I_2O_3 + NO_3^- \rightarrow IONO_2 + IO_3^-$. Given that $IONO_2$ signals do not exclusively originate from $IONO_2$ in NO_3^--CIMS, we believe the signals to predominately be measurement artefacts. $IONO_2$ signals are absent in Br^--CIMS. The quantitative and unambiguous detection of $IONO_2$ is likely facilitated by avoiding NO_3^-, and rather using e.g. Br^- as reagent ion.

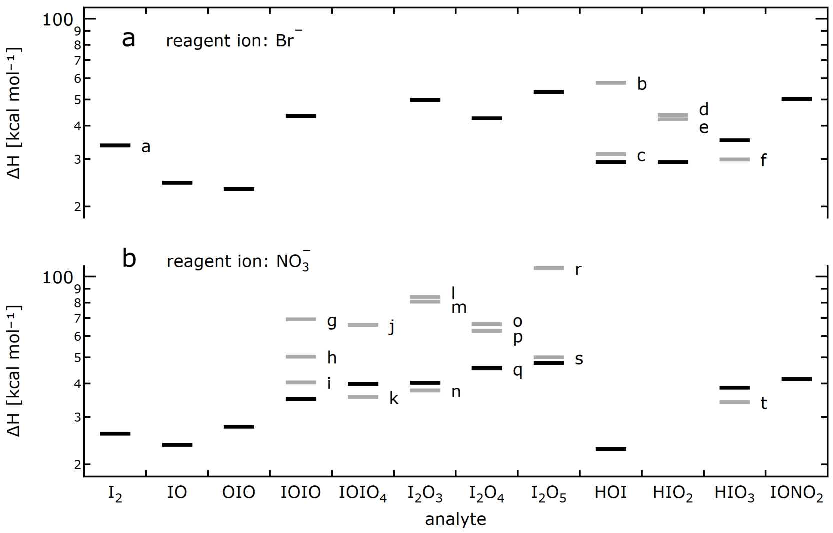

a: IBr + I^- (33.8). b: HBr + IO^- (57.7). c: HOBr + I^- (31.3). d: HBr + IO_2^- (43.8). e: HOBr + IO^- (42.2). f: HBr + IO_3^- (29.9).
g: $OIONO_2$ + IO^- (69.3). h: $IONO_2$ + OIO^- (50.3). i: $OIOONO_2$ + I^- (40.3). j: $OIONO_2$ + IO_4^- (66.0). k: $IONO_2$ + OIO_4^- (35.6).
l: $OIONO_2$ + OIO^- (83.8). m: I_2O_4 + NO_2^- (80.7). n: $IONO_2$ + IO_3^- (37.6). o: $OIOONO_2$ + OIO^- (66.4). p: I_2O_5 + NO_2^- (62.8).
q: $OIONO_2$ + IO_3^- (45.5). r: $IO_3NO_3^-$ + OIO^- (107.3). s: $OIOONO_2$ + IO_3^- (50.0). t: HNO_3 + IO_3^- (34.1).

Supplementary Fig. 3: Fragmentation enthalpies ΔH [kcal mol^{-1}] of reagent-ion–analyte adducts. Black lines show enthalpies for fragmentation into reagent ion and analyte, grey lines indicate fragmentation into other products (compare footnotes).

3 Quantum Chemical Calculations

3.1 Additional investigations on the fate of $IOIO_4$

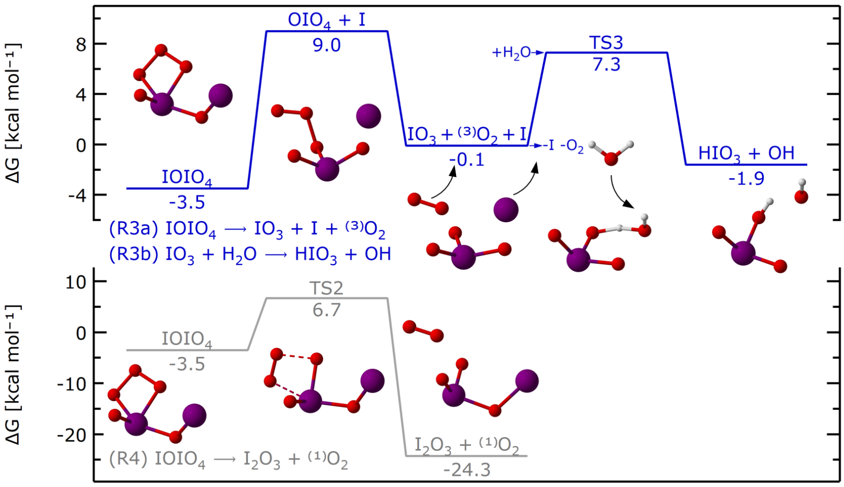

Supplementary Fig. 4: Reaction coordinate of alternative pathways. The energies are calculated using theory at the CCSD(T)/CBS(T,Q)//M062X/aug-cc-pVTZ-PP level.

Supplementary Table 4: Predicted energies and rates for reactions R3 and R4 using theory as in this study and as in the literature.

reaction	parameter	unit	theory[a] (literature)	theory[b] (this study)
(R3a) $IOIO_4$	ZPE	kcal mol^{-1}	20.6	22.5
$\rightarrow IO_3 + I + {}^{(3)}O_2$	G(298 K)	kcal mol^{-1}	1.4	3.4
(R3b) $IO_3 + H_2O$	ZPE	kcal mol^{-1}	4.4	-2.1
$\rightarrow HIO_3 + OH$	G(298 K)	kcal mol^{-1}	13.9	7.4
	k(298 K)	molec $cm^3\ s^{-1}$	$1.5 \cdot 10^{-17}$	$9.2 \cdot 10^{-13}$
	t(10^{17} molec cm^{-3} O_3)	s	0.6	$1.1 \cdot 10^{-5}$
(R4) $IOIO_4$	ZPE	kcal mol^{-1}	10.3	10.4
$\rightarrow I_2O_3 + {}^{(1)}O_2$	G(298 K)	kcal mol^{-1}	10.0	10.2
	k(298 K)	s^{-1}	$2.7 \cdot 10^5$	$2 \cdot 10^5$

[a] CCSD(T)/aug-cc-pVTZ+LANL2DZ//M062X/aug-cc-pVDZ+LANL2DZ, Gomez-Martin et al 2020, Kumar et al 2019, used in this work for comparison with literature.
[b] CCSD(T)/CBS(T,Q)//M062X/aug-cc-pVTZ-PP

We explored competing reactions of intermediate $IOIO_4$, specifically the decomposition into $IO_3 + I + {}^{(3)}O_2$ (R3a), and into $I_2O_3 + {}^{(1)}O_2$ (R4). The associated reaction coordinate is shown in Supplementary Fig. 4, calculated energies are shown in Supplementary Table 4.

For reaction R3a, the coupled-cluster calculations on the intermediates and TS, particularly OIO_4 and TS3, show high T1 diagnostic numbers (0.046 and 0.037, respectively), and the predicted energies and rate constants are consequently highly unreliable. IO_3 is predicted to react reasonably fast with water ($k = 9.2 \cdot 10^{-13}\,\mathrm{molec\,cm^3\,s^{-1}}$) to form HIO_3 and OH radicals (R3b), resulting in a rapid conversion even at moderate water concentrations. This is somewhat in contrast to expectations in the literature that this reaction would be prohibitively slow [18, 25]. Consequentially, reactions R3a and R3b could in principle be additional pathways to HIO_3 and HOI (OH reacts rapidly with I_2 to form HOI, and HO_2 reacts rapidly IO to form HOI), and would be compatible with experiments (Supplementary Table 2 and Extended Data Fig. 2), but are not needed to explain the observations. Theory, as used in this study, does not find evidence that reaction R3a is feasible, and there is no firm experimental evidence that R3a occurs. Reaction R2 is recommended. Future experiments could make an attempt to detect the side products of reaction R2, singlet oxgyen, and R3b, OH.

For reaction R4, the transition state shows a similarly high T1 diagnostic of 0.031, making the predicted energies and rate constant highly uncertain. If feasible, this reaction would compete against the formation of HIO_3. Given that the sensitivity of NO_3^--CIMS to I_2O_3 detection should be significant (Supplementary Fig. 3), the absence of I_2O_3 in measurements supports that reaction R4 is not happening. We therefore conclude that reaction R4 is not occurring at a significant rate.

3.2 Sensitivity studies

Wavefunction stability

The stability of the wavefunction was checked at the CCSD(T) stage by first running HF calculations with 15 HOMOs and 15 LUMOs switched 10 times randomly and generating 100 input files with the orbital rotations applied [26]. This indicated that no lower-lying wavefunction relative to the default solution was neglected for any of the intermediates and transition states along the I_2O_2 + O_3 PES. The HF calculations were carried out with the def2-QZVPP basis set and using the ORCA version 4.2.1 program. This is a much more robust approach than e.g. the standard Stable=Opt check in Gaussian.

Spin-orbit coupling correction

The spin-orbit coupling corrections of each species along the I_2O_2 + O_3 PES and the difference in corrections between different stationary points are provided in Supplementary Table 5 and 6. Note that the effective-core potentials for the iodine atom used in the DFT and CCSD(T) calculations already include some fraction of SOC, and the actual correction to the energies in Fig. 3 will be less than what the table indicates.

Supplementary Table 5: Spin-orbit coupling energies of the I_2O_2 + O_3 reaction stationary points.

molecule	spin-orbit energy [E_h]
I_2O_2	-9.74
O_3	0.00
TS1	-9.74
$IOIO_4$	-9.73
TS2	-9.73
IOIOOHOOOH	-9.73
TS3	-9.73
H_2O	0.00

Supplementary Table 6: Relative spin-orbit coupling energies.

reaction	relative spin-orbit energy kcal mol^{-1}
$I_2O_2 + O_3 \rightarrow$ TS1	1.2
$I_2O_2 + O_3 \rightarrow IOIO_4$	3.1
$I_2O_2 + O_3 + H_2O \rightarrow$ TS2	2.9
$I_2O_2 + O_3 + H_2O \rightarrow$ IOIOOHOOOH	3.7
$I_2O_2 + O_3 + H_2O \rightarrow$ TS3	3.6

$^{(3)}O_2$ / $^{(1)}O_2$ gap

The reliability of the selected level of theory was also checked by calculating the $^{(3)}O_2$ / $^{(1)}O_2$ energy gap = 29.7 kcal mol^{-1}, which is ~7 kcal mol^{-1} higher than the experimental value of 22.6 kcal mol^{-1}. Note that computing the $^{(3)}O_2$ / $^{(1)}O_2$ energy gap is a well-known failure for almost all affordable QC methods. It would be necessary to use methods such as CCSDTQ to reproduce the energy gap accurately, but this method is not affordable for molecules larger than O_2.

Quasi-harmonic treatment

A quasi-harmonic approximation [27] was implemented on frequencies below 100 cm^{-1} for all molecules along the $I_2O_2 + O_3$ reaction PES to evaluate the influence of internal rotations on the energetics. The difference in energies were less than 0.02 kcal mol^{-1}.

3.3 Guidance for model development

IOIO lifetime

O_3 decay ramps at 263 K find indirect experimental evidence in support of the longer IOIO lifetime predicted by quantum chemical calculations in this study compared to the literature (compare Table 1). This is because the fate of IOIO to react with O_3 is in competition with either the thermal decomposition or the wall loss. The current literature suggests the IOIO thermal lifetime to form OIO and I is ~100 s at 263 K. If this was correct, thermal decomposition would be the rate limiting sink for IOIO at the lower end of the O_3 concentrations probed at CLOUD. Supplementary Figure 5 shows the lifetimes of IOIO in regard to different loss mechanisms. Experimentally, no significant deviation from the quantitative conversion of IOIO into HIO_3 is observed even under these extreme conditions (Extended Data Fig. 4). The experimentally inferred value of k_1 in Table 1 is therefore estimated conservatively as lower limit.

Rate constants k_1 and k_2 for atmospheric modelling purposes

The experimental rate constant, using the temperature dependence predicted by theory, is $k_1 = 8.2 \cdot 10^{-15} \cdot \exp(763\,\mathrm{K}/T)$ molec $cm^3\,s^{-1}$. The laboratory experiments provide no strong experimental constraint on k_2. The best estimate based on theory, compatible with laboratory experiments and field measurements, is $k_2 = 2.5 \cdot 10^{-12} \cdot \exp(-2481\,\mathrm{K}/T)$ molec $cm^3\,s^{-1}$. Supplementary Figure 6 suggests that k_1 will accelerate as temperature decreases, consistent with the expectation for an O_3 addition reaction to IOIO. The rate coefficient k_2 slows down faster as temperature decreases, and this is further compounded by generally lower water concentrations at lower temperatures. No net-effect of temperature is observed over the limited temperature range probed in this study. More studies are needed to establish whether either O_3 or H_2O could become limiting to the production of HIO_3 in extremely cold, dry and low O_3 atmospheric environments, e.g., in the upper troposphere – lower stratosphere.

Note: Treating bimolecular reactions of an intermediate carrying excess energy in master equation simulations is non-trivial. For the $IOIO_4 + H_2O$ step in the present case, the MesmerILT method was used

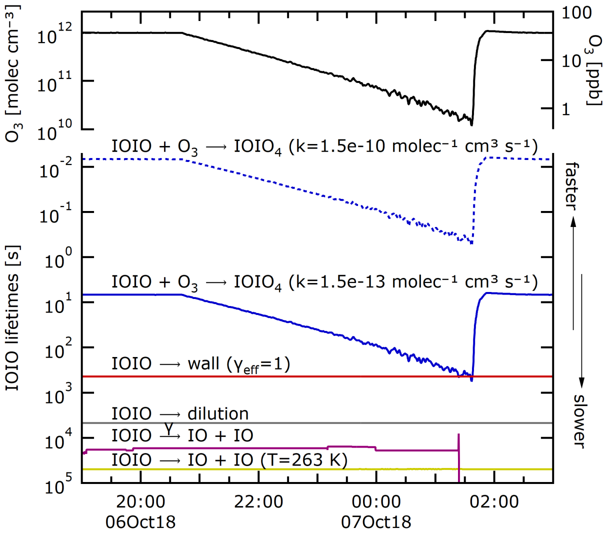

Supplementary Fig. 5: Sinks of IOIO during variations of O_3, at $T = 263\,\mathrm{K}$. Thermal decomposition IOIO $\rightarrow$ OIO + I is likely overestimated in the current literature. This study finds that IOIO thermal decomposition is not significant under the conditions probed, and the reaction with O_3 is the main sink for IOIO. The blue dashed line indicates the fate of IOIO if the reaction with O_3 proceeded near the kinetic limit.

with a defined activation energy corresponding to the quantum chemically calculated barrier to directly lead to the intermediate IOIOOHOOOH. A similar method was used previously by Shannon et al. [28] to treat their reaction HC(O)C(O) + O_2. We do note that $IOIO_4$ + H_2O forms a pre-reactive complex $IOIO_4 \cdot H_2O$ that is $\sim$10 kcal mol^{-1} below the reactants in zero-point corrected energies. Accounting for the partitioning of the excess energy in $IOIO_4 \cdot H_2O$ can change the MESMER derived temperature dependent rate $k_2(T)$. Indeed, a simulation that includes the pre-reactive complex results in a $k_2(298\,\mathrm{K})$ of $\sim 8 \cdot 10^{-19}\,\mathrm{molec\,cm^3\,s^{-1}}$, which is 2 to 3 orders of magnitude slower than the reported theoretical value in Table 1. Not surprisingly, there are significant uncertainties in estimating the bimolecular rate coefficients of an intermediate carrying excess energy. However, the experimental constraint of $k_2(263\,\mathrm{K})$ in Table 1 adds credence for the involvement of hot $IOIO_4$ in R2, and is consistent with the MesmerILT method; together, the experimental and theoretical evidence support the reported rate coefficient at 263 K, but k_2(T) warrants further investigation.

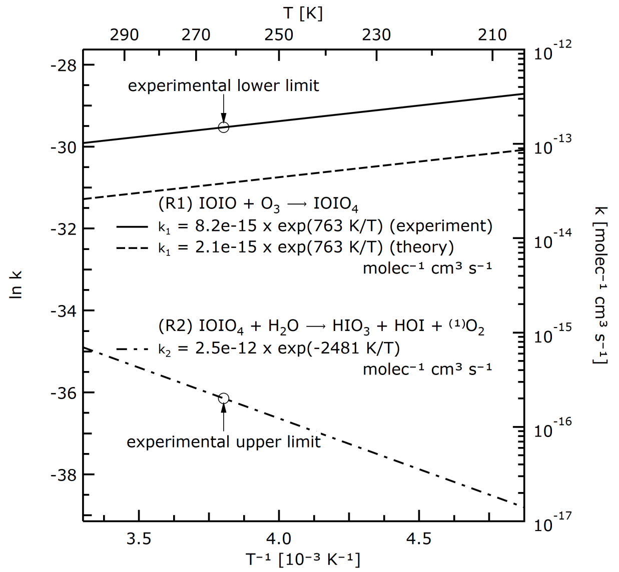

Supplementary Fig. 6: Temperature dependent rate constants for reactions R1 and R2. The experimentally derived k_1 is ~ 4 times larger than predicted by theory, well within the uncertainty associated with the calculations.

4 Maïdo field measurements

The Maïdo observatory is located on the western slope of Réunion island in the southern Indian Ocean (21.1° S, 55.4° E). At an elevation of of 2160 m asl the observatory provides frequent access to lower free tropospheric air at night and during the early morning. South-easterly trade winds prevail in the area. Frequently, the heating of the island locally initiates a coastal anabatic wind a few hours after sunrise, and the wind direction at the observatory shifts from south-easterly to westerly (e.g., Supplementary Fig. 7, Apr 13, 04:30 UTC). As a consequence, the origin of air masses sampled during different periods of the day can vary typically in the mid and later morning. Several proxies for the air mass origin are sampled at the observatory: Radon (boundary layer influence), NO_2 (human activity), isoprene (bio-activity of adjacent forest), sulfuric acid (human activity and emissions of the adjacent volcano, Piton de la Fournaise), along with basic meteorology. Temperature T, dew point temperature T_{dew} and wind direction are given in Supplementary Fig. 7 to illustrate the constancy of air masses during the modelled period. Figure 4 is derived by assuming steady state between HIO_3 production and loss to particles at every point in time. It displays all data with modelled [IO] $> 10^6$ molec cm^3 (day-time conditions) collected during the field campaign, to increase the number of data points. Data stringently filtered for free tropospheric origin fall into the scatter. This suggests that the influence of contamination to HIO_3 formation is likely limited for the probed conditions.

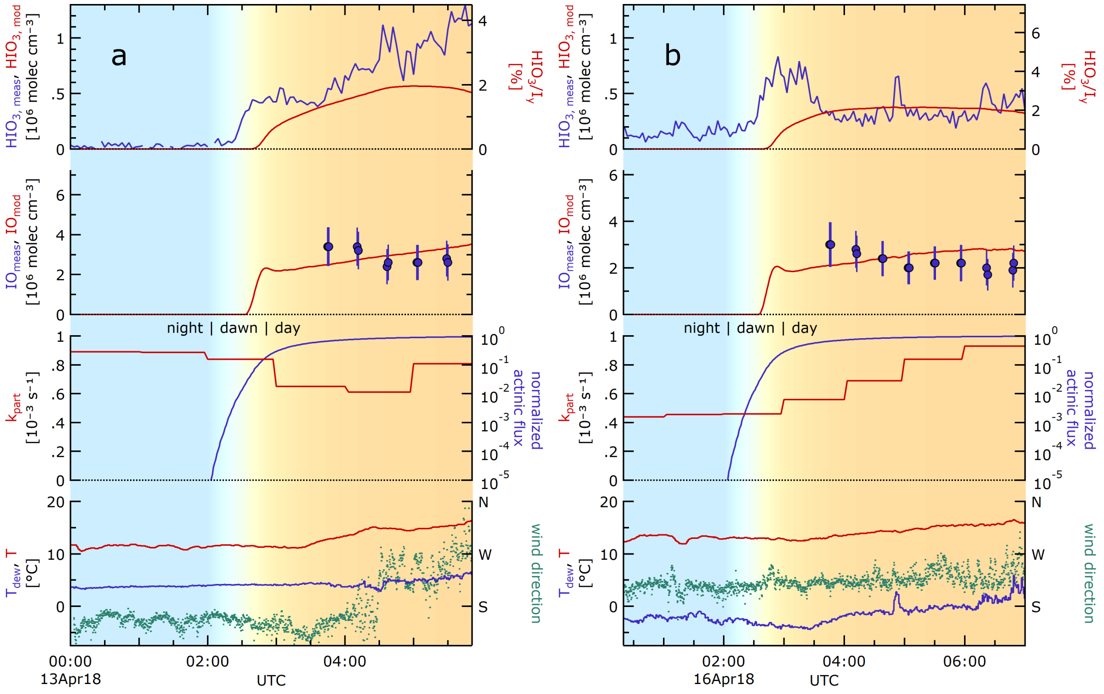

Supplementary Fig. 7: Detection of iodine species at the Maïdo field site, Réunion Island, 2200 m ASL, southern Indian Ocean for two different days (a and b). Background colours indicate night, dawn, and day. HIO_3 concentrations measured and modelled (left axis), modelled fraction of HIO_3 in total I_y budget (right axis), IO as measured by MAX-DOAS and used in model (error bars are 30 % (2-sigma, 95 % CI), see [29]), condensation rate to particles, normalised actinic flux, temperature, dew point temperature and wind direction as proxy for air mass variability.

4.1 Chemical box modelling

Chemical box modelling for the Maïdo field site employs the same reactions as for the CLOUD laboratory, but is extended by NOx chemistry (Supplementary Table 9). It is constrained by measurements of temperature, pressure, humidity, IO concentrations, O_3 concentrations, loss of HIO_3 to particle surface area. Integral measurements of actinic fluxes are available at the observatory, and indicate cloud free mornings; for the calculations of compound specific photolysis frequencies actinic fluxes determined by TUV [30] were used. NOx was fixed to 10^9 molec cm^{-3} (50 pptv).

HIO_3 is lost to particles (typical condensation sink rate 10^{-3} s^{-1}). As HIO_3 is typically the third most abundant iodine species ([HIO_3] : [IO] : [HOI] ≈ 2 % : 15 % : 80%), condensation of I_xO_y to particles is likely a minor contribution and does not substantially change the partitioning. HIO_3 is subsequently re-emitted into the gas phase as HOI (main iodine reservoir), to maintain the total I_y (inferred from IO radical observations, and the IO/Iy,gas ratio) during a simulation. The model assumes the re-emission to be instantaneous. As long as the re-emission time is significantly faster than the condensation time, there is little sensitivity to the resulting gas-phase iodine partitioning. It is almost certain that the recycling time will vary for different conditions (e.g., day, night, dusk and dawn), and there is a need to elucidate the governing processes quantitatively at a molecular level.

4.2 Modelled HIO_3 time series

Supplementary Figure 7 shows time series predicted by the extended model, and compares them to measurements. The two mornings are displayed here, because cloud free and stable morning conditions provide access to the lower free troposphere, and because of the availability of CU MAX-DOAS IO radical measurements to constrain total I_y in the extended model simulation. The shading indicates the night–dawn–day transition (also shown as normalised actinic flux). The bottom panel shows temperature T, dew-point temperature T_{dew} and the wind direction as indicator for the variability of conditions. The extended model reproduces day-time concentrations of HIO_3 within the uncertainty of the environment.

HIO_3 is formed already during the dawn. Very little light is required to initiate its formation. The formation *under cloudy daylight conditions with negligible ultraviolet irradiation* has been noted previously [1]. The measurements even slightly precede the model prediction. This could be explained by the activation of night-time iodine reservoirs [31] at first light. Supplementary Figure 7B even shows some HIO_3 production during night. This observation is consistent with previous studies [24, 32, 33] and indicative that active iodine chemistry can form some HIO_3 also at night-time.

4.3 Modelled $I_{y,\mathrm{gas}}$ partitioning

Supplementary Figure 8 shows time series for the predicted $I_{y,\mathrm{gas}}$ partitioning for the case study days shown in Supplementary Fig. 7. The total iodine burden $I_{y,\mathrm{gas}}$ is constrained by measurements of IO radicals, and box modelling that either uses the model base case (A and C) or the extended model (B and D, forms HIO_3). The HIO_3 / $I_{y,\mathrm{gas}}$ ratio is determined by measurements of HIO_3 (solid red line); or the predicted iodine species (dashed lines); the sum of predicted I_xO_y ($x \geq 2$, $y \geq 2$) is further shown (solid blue line).

As can be seen, the I_xO_y concentrations are not sufficient to explain HIO_3 concentrations under the probed conditions, independent of the model used, lending further support from a mass balance perspective that there is insufficient amounts of I_xO_y formed to explain HIO_3 as a measurement artefact (see Supplementary Section 2.3). Note that both models conservatively estimate the I_xO_y / $I_{y,\mathrm{gas}}$ ratio here, given the extended model overestimates I_xO_y species compared to the laboratory measurements at CLOUD (Fig. 1 and Extended Data Fig. 3), and because the added HIO_3 formation in the extended model directly competes with I_xO_y formation by deviating the oxidation pathways following IOIO. Even if all [I_xO_y] was detected as HIO_3, the measured concentrations of HIO_3 would be essentially unexplained.

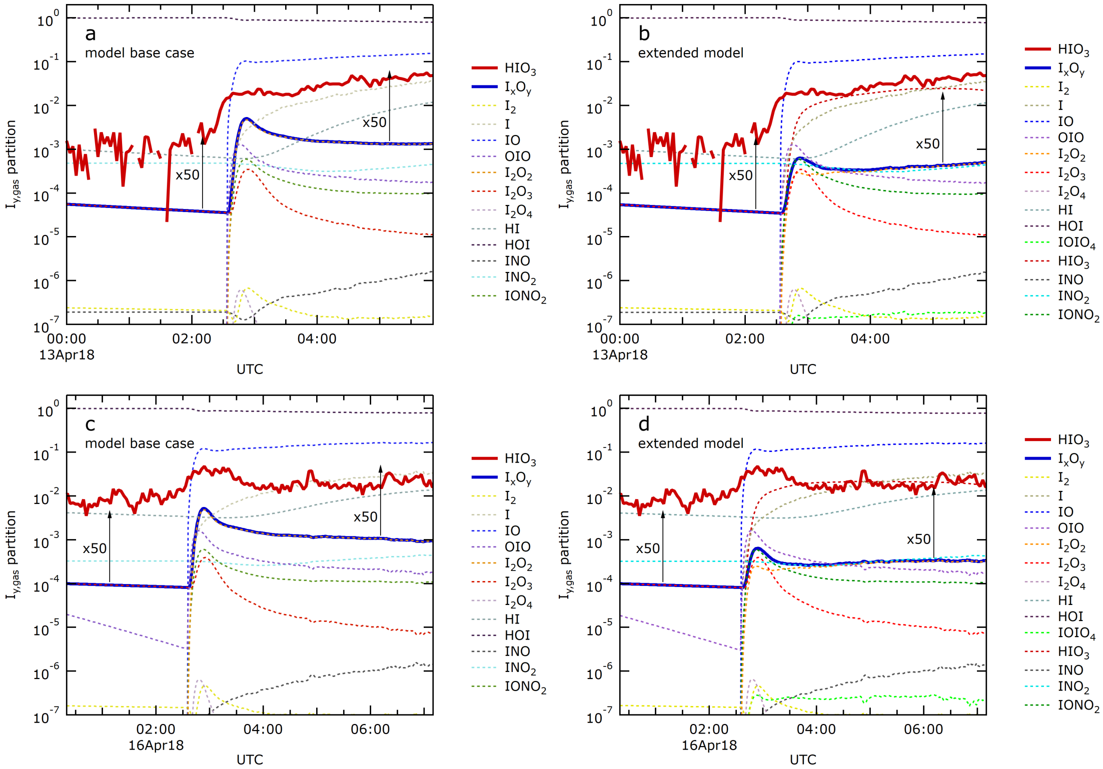

Supplementary Fig. 8: Iodine partitioning at the Maïdo field site, showing $[I_xO_y] \ll [HIO_3]$. The total iodine burden I_y is constrained by measurements of IO radicals, and box modelling that either uses the model base case (a and c) or the extended model (b and d, forms HIO_3). The HIO_3 / $I_{y,gas}$ ratio is determined by measurements of HIO_3 (solid red line); the partition of other iodine iodine species and sum I_xO_y ($x \geq 2$, $y \geq 2$) (solid blue line) is predicted by box-modelling.

5 HIO_3 formation in flow-tube experiments

5.1 Sensitivity of HIO_3 formation to humidity

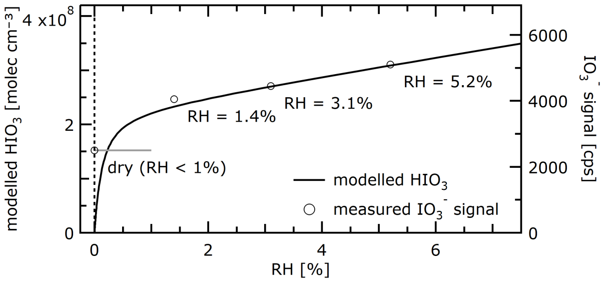

Supplementary Fig. 9: Formation of HIO_3 at variable humidity in flow tube as predicted by extended mechanism in Sipilä type flow tube [9]. Below a few percent relative humidity a strong humidity sensitivity is observed and predicted, which then gets reduced.

Sipilä et al. [16] had noted previously a sensitivity of HIO_3 formation (observed as IO_3^-) to humidity in flow tube experiments: At very low humidities (low single % relative humidity) HIO_3 production was found to be reduced, at higher humidity only a weak sensitivity was observed (Supplementary Fig. 9, open circles). We can apply the extended model including the mechanism to explain the behaviour.

We approximate the conditions of the underlying experiment, for which more accurate descriptions are not available, using the following parameters: Measurements were carried out at room air temperature: $T = 293\,\mathrm{K}$. A flow tube of 1 m length and 5 cm diameter results in a volume of 2 l. At a flow rate 13.5 l min^{-1} this is equivalent to a residence time of 9 s. A mercury lamp was used, for which we assume a similar spectrum as UVH at CLOUD (Supplementary Fig. 10), with an effective I_2 photolysis frequency $j(I_2) = 1.5 \cdot 10^{-2}\,\mathrm{s}^{-1}$. Further, we assume $[I_2] = 2.5 \cdot 10^{10}$ molec cm^{-3} (1 ppbv), $[O_3] = 1 \cdot 10^{12}$ molec cm^{-3} (40 ppbv). Accommodation losses to the flow tube walls are considered to occur on time scales much longer than the residence time, but should regardless not critically influence the results. The predicted HIO_3 concentrations after 9 s of transport in the flow tube are shown in Supplementary Fig. 9 together with measured HIO_3 signals. The extended model reproduces the observed trend.

The slight HIO_3 increase above 1 % relative humidity is explained in the model by an increasingly relevant production of OH radicals from $O(^1D) + H_2O$ at high humidity, that reacts with I_2 to release more I radicals. The very strong sensitivity at $< 1\%$ relative humidity is explained by the low rate of conversion of $IOIO_4$ by water. Under these conditions with $[IO] \approx 10^9$ molec cm^{-3}, water ceases to be the limiting reagent at $\sim 1\%$ relative humidity ($6 \cdot 10^{15}$ molec cm^{-3}). At higher IO concentrations, i.e., higher production rates of IOIO and $IOIO_4$, progressively higher concentrations of water would be required to appreciably convert $IOIO_4$ into HIO_3. Assuming a quadratic dependency, an increase of $[IO] \approx 10^9$ molec cm^{-3} to $[IO] \approx 10^{12}$ molec cm^{-3} (factor 10^3), the critical relative humidity would increase by a factor 10^6, from $\sim 1\%$ to 10^4 times the saturation vapor pressure. In other words, water is necessarily a limiting reagent to HIO_3 formation under extremely high IO_x concentrations, and HIO_3 could not form as significant product.

5.2 Competition of HIO_3 & I_xO_y in flow tube experiments

Gomez-Martin et al. [2] studied iodine particle formation from larger I_xO_y in flow tube experiments, and did not detect HIO_3. We believe this observation is consistent with the proposed mechanism.

Typical IO radical concentrations in these flow tube experiments (compare Fig. 4 in [2] are given in Supplementary Table 1 (flow tube), and strongly favour the formation of large iodine oxide cluster through

polymerisation. HIO_3 is not expected to form in appreciable amounts, because water concentrations cannot be increased as much as iodine concentrations. Gomez-Martin et al. [2] did not report a sensitivity towards HIO_3/IO_3^- for the photo-ionisation technique used to detect iodine oxides. The authors suggested that IO_3^-, interpreted as HIO_3, forms in the fragmentation of larger I_xO_y species upon detection, i.e., chemical ionisation [2]. We have high trust in the real character of HIO_3, given that there is a viable gas-phase mechanism to form it, and because it is measured in parallel by multiple instruments that employ different parameters and ionisation techniques: NO_3^--CIMS, Br^--MION-CIMS, HOxROx-NO_3^--CIMS, APiTOF (no ionisation). Theory as in this study predicts that I_2O_3 as early generation I_xO_y should be detectable by NO_3^--CIMS (Supplementary Section 2.4), but I_2O_3 is not observed as major iodine reservoir. Additionally, the appearance of I_xO_y ($x \geq 2$, $y \geq 3$) is too late to be compatible with the fast appearance of HIO_3 (Fig. 1, Extended Data Fig. 2, Supplementary Table 2). This study thus supports that HIO_3 in the gas phase is a real and abundant species.

An early study [34] had found a negative correlation at a coastal site between the frequency of particle formation and water vapour flux and relative humidity, and had interpreted this as *some support for the production of new particles through the self-nucleation of iodine oxides proposed by Hoffmann et al. [2001]*. One plausible explanation for this behaviour is that *H_2O forms relatively stable complexes with molecules such as I_2O_3 and I_2O_4, inhibiting their polymerization* [35], and the unusual hygroscopic growth behaviour of iodine oxide particles in laboratory studies has also been noted [36, 37]. Under near-atmospheric conditions at CLOUD, nucleation rates are essentially independent of humidity [1]. An inhibiting role of water may be relevant for conditions with high I_xO_y [2], but such a role of water is neither observed at CLOUD, nor is iodine particle formation inhibited at very high humidity in the arctic marine boundary layer (median 95.7 % relative humidity, see [24]).

6 Description of the chemical box-model

The photochemical box model builds on a framework described in [3, 4, 38] and represents state-of-the-art iodine chemistry and HO_x chemistry [39, 40]. Here, the model is extended by the chamber auxiliary mechanism, which includes losses of gases to the chamber walls and to particles, losses by dilution, and the actinic fluxes of the chamber lights. The model is constraint by measurements of I_2, O_3, H_2O, photolysis frequencies (I_2, IO, OIO, HOI, I_2O_2, I_2O_3, I_2O_4), temperature, and aforementioned loss mechanisms.

6.1 Gas-phase reactions

Iodine gas-phase reactions are taken from [39], where recommendations are available. Reaction rate constant expressions for the recombination of early iodine oxides are taken from a recent literature review [41]. The dark reaction of I_2 with O_3 [42] has recently been corroborated theoretically [43], and is included here. HO_x reactions, particularly relevant for the description of the chemistry at the Maïdo field site, are

Supplementary Table 7: Gas-phase iodine reactions used in model base case.

Reaction	k [molec^{-1} cm^3 s^{-1}]	notes
$I_2 + O \rightarrow IO + I$	$1.3 \cdot 10^{-10}$	[39]
$I + O_3 \rightarrow IO + O_2$	$2.0 \cdot 10^{-11} \cdot \exp(-830/T)$	[39]
$IO + O \rightarrow I + O_2$	$1.4 \cdot 10^{-10}$	[39]
$IO + O_3 \rightarrow OIO + O_2$	$3.6 \cdot 10^{-16}$	[44]
$IO + IO \rightarrow OIO + I$	$2.13 \cdot 10^{-11} \cdot \exp(180\,K/T)$ $\cdot(1 + \exp(-p/19142\,Pa))$	[41]
$IO + IO \rightarrow IOIO$	$3.27 \cdot 10^{-11} \cdot \exp(180\,K/T)$ $\cdot(1 - 0.65 \cdot \exp(-p/19142\,Pa))$	[41]
$IO + OIO \rightarrow I_2O_3$	[a]	[41]
$OIO + OIO \rightarrow I_2O_4$	[b]	[41]
$I_2 + OH \rightarrow HOI + I$	$1.8 \cdot 10^{-10}$	[39]
$HOI + OH \rightarrow IO + H_2O$	$2.0 \cdot 10^{-13}$	[45, 46]
$IO + OH \rightarrow HO_2 + I$	$1.0 \cdot 10^{-10}$	[47]
$IO + HO_2 \rightarrow HOI$	$1.3 \cdot 10^{-11} \cdot \exp(570\,K/T)$	[39][c]
$I + HO_2 \rightarrow HI + O_2$	$1.5 \cdot 10^{-11} \cdot \exp(-1090\,K/T)$	[39]
$HI + OH \rightarrow I + H_2O$	$3.0 \cdot 10^{-11}$	[39]
$I_2 + O_3 \rightarrow IO + OIO$	$0.5 \cdot 4.0 \cdot 10^{-15} \cdot \exp(-2050\,K/T)$	[42, 43][d]
$I_2 + O_3 \rightarrow IO + I + O_2$	$0.5 \cdot 4.0 \cdot 10^{-15} \cdot \exp(-2050\,K/T)$	[42, 43][d]

[a] $k = (4.687 \cdot 10^{-10} - 1.3855 \cdot 10^{-5} \cdot \exp(-0.75p/162.265\,Pa) + 5.51868 \cdot 10^{-10} \cdot \exp(-0.75p/19932.8\,Pa)) \cdot \exp((-3.31 \cdot 10^{-3} - 5.14 \cdot 10^{-3} \cdot \exp(-0.75p/32568.711\,Pa) - 4.44 \cdot 10^{-3} \cdot \exp(-0.75p/4081.609\,Pa)) \cdot T)$

[b] $k = (1.1659 \cdot 10^{-9} - 7.79644 \cdot 10^{-10} \cdot \exp(-0.75p/2209.281\,Pa) + 1.03779 \cdot 10^{-9} \cdot \exp(-0.75p/56815.381\,Pa)) \cdot \exp((-8.13 \cdot 10^{-3} - 3.82 \cdot 10^{-3} \cdot \exp(-0.75p/4557.591\,Pa) - 6.43 \cdot 10^{-3} \cdot \exp(-0.75p/41795.061\,Pa)) \cdot T)$

[c] HOI assumed to be only product

[d] products not clear, equal branching assumed

taken from [39], and listed in Supplementary Table 8. NOx chemistry is taken from [39] unless otherwise noted. While this study does not leverage laboratory data that involve NOx, NOx is relevant for night-time chemistry in the field. It is therefore approximated with the reactions in Supplementary Table 9.

Supplementary Table 8: HO_x reactions in photochemical box model, taken from [39]

Reaction	k [molec^{-1} cm^3 s^{-1}]
$O + O_2 \rightarrow O_3$	$6.1 \cdot 10^{-34} \cdot (T/298\,K)^{-2.4} \cdot [air]$
$O + O_3 \rightarrow O_2 + O_2$	$8.0 \cdot 10^{-12} \cdot \exp(-2060\,K/T)$
$O(^1D) + N_2 \rightarrow O(^3P) + N_2$	$2.15 \cdot 10^{-11} \cdot \exp(110\,K/T)$
$O(^1D) + O_2 \rightarrow O(^3P) + O_2$	$3.3 \cdot 10^{-11} \cdot \exp(55\,K/T)$
$O(^1D) + O_3 \rightarrow O_2 + O_2$	$0.5 \cdot 2.4 \cdot 10^{-10}$
$O(^1D) + O_3 \rightarrow O(^3P) + O(^3P) + O_2$	$0.5 \cdot 2.4 \cdot 10^{-10}$
$O(^1D) + H_2 \rightarrow OH + H$	$1.2 \cdot 10^{-10}$
$O(^1D) + H_2O \rightarrow OH + OH$	$1.63 \cdot 10^{-10} \cdot \exp(60\,K/T)$
$O(^1D) + N_2 \rightarrow N_2O$	$2.8 \cdot 10^{-36} \cdot (T/300\,K)^{-0.9} \cdot [air]$
$O + OH \rightarrow H + O_2$	$1.8 \cdot 10^{-11} \cdot \exp(180\,K/T)$
$O + HO_2 \rightarrow OH + O_2$	$3.0 \cdot 10^{-11} \cdot \exp(200\,K/T)$
$O + H_2O_2 \rightarrow OH + HO_2$	$1.4 \cdot 10^{-12} \cdot \exp(-2000\,K/T)$
$H + O_2 \rightarrow HO_2$	$k_f{}^{ab}$
$H + O_3 \rightarrow OH + O_2$	$1.4 \cdot 10^{-10} \cdot \exp(-470\,K/T)$
$H + HO_2 \rightarrow OH + OH$	$7.2 \cdot 10^{-11}$
$H + HO_2 \rightarrow O + H_2O$	$1.6 \cdot 10^{-12}$
$H + HO_2 \rightarrow H_2 + O_2$	$6.9 \cdot 10^{-12}$
$OH + O_3 \rightarrow HO_2 + O_2$	$1.7 \cdot 10^{-12} \cdot \exp(-940\,K/T)$
$OH + H_2 \rightarrow H + H_2O$	$2.8 \cdot 10^{-12} \cdot \exp(-1800\,K/T)$
$OH + OH \rightarrow O + H_2O$	$1.8 \cdot 10^{-12}$
$OH + OH \rightarrow H_2O_2$	$k_f{}^{ac}$
$OH + H_2O_2 \rightarrow HO_2 + H_2O$	$1.8 \cdot 10^{-12}$
$OH + HO_2 \rightarrow H_2O + O_2$	$4.8 \cdot 10^{-11} \cdot \exp(250\,K)$
$HO_2 + O_3 \rightarrow OH + O_2 + O_2$	$1.0 \cdot 10^{-14} \cdot \exp(-490\,K)$
$HO_2 + HO_2 \rightarrow H_2O_2 + O_2$	$3.0 \cdot 10^{-13} \cdot \exp(460\,K)$
$HO_2 + HO_2 \rightarrow H_2O_2 + O_2$	$2.1 \cdot 10^{-33} \cdot \exp(920\,K) \cdot [air]$
$OH + CO \rightarrow HO_2 + CO_2$	$1.85 \cdot 10^{-13} \cdot \exp(-65\,K/T)$

[a] effective second-order rate constant $k_f(T,[M])$ as defined in [39]
[b] $k_0 = 5.3 \cdot 10^{-32}$, $n = 1.8$, $k_\infty = 9.5 \cdot 10^{-11}$, $m = -0.4$, $[M] = [air]$
[c] $k_0 = 6.9 \cdot 10^{-31}$, $n = 1.0$, $k_\infty = 2.6 \cdot 10^{-11}$, $m = 0$, $[M] = [air]$

Supplementary Table 9: Gas-phase NOx reactions used in model. Taken from [39] unless otherwise noted.

Reaction	k [molec^{-1} cm^3 s^{-1}]	notes
$I + NO_3 \rightarrow IO + NO_2$	$4.5 \cdot 10^{-10}$	[48]
$I_2 + NO_3 \rightarrow I + IONO_2$	$1.5 \cdot 10^{-12}$	[48]
$IONO_2 + I \rightarrow I_2 + NO_3$	$1 \cdot 10^{-10}$	estimated, [49]
$I + NO \rightarrow INO$	$k_f{}^{ab}$	
$I + NO_2 \rightarrow INO_2$	$k_f{}^{ac}$	
$IO + NO \rightarrow I + NO_2$	$8.6 \cdot 10^{-12} \cdot \exp(230\,K/T)$	
$IO + NO_2 \rightarrow IONO_2$	$k_f{}^{ad}$	
$INO + INO \rightarrow I_2 + NO + NO$	$8.4 \cdot 10^{-11} \cdot \exp(-2620\,K/T)$	
$INO_2 + INO_2 \rightarrow I_2 + NO_2 + NO_2$	$2.9 \cdot 10^{-11} \cdot \exp(-2600\,K/T)$	
$O + NO \rightarrow NO_2$	$k_f{}^{ae}$	
$O + NO_2 \rightarrow NO + O_2$	$5.3 \cdot 10^{-12} \cdot \exp(200\,K/T)$	
$O + NO_2 \rightarrow NO_3$	$k_f{}^{af}$	
$O + NO_3 \rightarrow NO_2 + O_2$	$1.3 \cdot 10^{-11}$	
$O + HNO_3 \rightarrow OH + NO_3$	$3.0 \cdot 10^{-17}$	upper limit
$H + NO_2 \rightarrow OH + NO$	$1.35 \cdot 10^{-10}$	
$OH + NO \rightarrow HONO$	$k_f{}^{ag}$	
$OH + NO_2 \rightarrow HNO_3$	$k_f{}^{ah} + k_f{}^{ai}$	2 isomer channels
$OH + NO_3 \rightarrow HO_2 + NO_2$	$2.0 \cdot 10^{-11}$	
$OH + HNO_3 \rightarrow NO_3 + H_2O$	$3.7\dot{\imath}10^{-14} \cdot \exp(240\,K/T)$	
$OH + HO_2NO_2 \rightarrow NO_2 + H_2O + O_2$	$4.5 \cdot 10^{-13} \cdot \exp(610\,K/T)$	
$HO_2 + NO \rightarrow NO_2 + OH$	$3.44 \cdot 10^{-12} \cdot \exp(260\,K/T)$	
$HO_2 + NO_2 \rightarrow HO_2NO_2$	$k_f{}^{aj}$	
$HO_2 + NO_2 \rightarrow HONO + O_2$	$5 \cdot 10^{-16}$	upper limit
$HO_2 + NO_3 \rightarrow OH + NO_2 + O_2$	$3.5 \cdot 10^{-12}$	
$NO + O_3 \rightarrow NO_2 + O_2$	$3.0 \cdot 10^{-12} \cdot \exp(-1500\,K/T)$	
$NO + NO_3 \rightarrow NO_2 + NO_2$	$1.7 \cdot 10^{-11} \cdot \exp(125\,K/T)$	
$NO_2 + O_3 \rightarrow NO_3 + O_2$	$1.2 \cdot 10^{-13} \cdot \exp(-2450\,K/T)$	
$NO_2 + NO_3 \rightarrow NO + NO_2 + O_2$	$4.35 \cdot 10^{-14} \cdot \exp(-1335\,K/T)$	
$NO_2 + NO_3 \rightarrow N_2O_5$	$k_f{}^{ak}$	
$NO_3 + NO_3 \rightarrow NO_2 + NO_2 + O_2$	$8.5 \cdot 10^{-13} \cdot \exp(-2450\,K/T)$	
$O_3 + HNO_2 \rightarrow HNO_3 + O_2$	$5.0 \cdot 10^{-19}$	upper limit
$N_2O_5 + H_2O \rightarrow HNO_3 + HNO_3$	$2.0 \cdot 10^{-21}$	upper limit

[a] effective second-order rate constant $k_f(T, [M])$ as defined in [39]
[b] $k_0 = 1.8 \cdot 10^{-32}$, $n = 1.0$, $k_\infty = 1.7 \cdot 10^{-11}$, $m = 0$, $[M] = [\text{air}]$
[c] $k_0 = 3.0 \cdot 10^{-31}$, $n = 1.0$, $k_\infty = 6.6 \cdot 10^{-11}$, $m = 0$, $[M] = [\text{air}]$
[d] $k_0 = 7.7 \cdot 10^{-31}$, $n = 3.5$, $k_\infty = 7.7 \cdot 10^{-12}$, $m = 1.5$, $[M] = [\text{air}]$
[e] $k_0 = 9.1 \cdot 10^{-32}$, $n = 1.5$, $k_\infty = 3.0 \cdot 10^{-11}$, $m = 0$, $[M] = [\text{air}]$
[f] $k_0 = 2.5 \cdot 10^{-31}$, $n = 1.8$, $k_\infty = 2.2 \cdot 10^{-11}$, $m = 0.7$, $[M] = [\text{air}]$
[g] $k_0 = 7.1 \cdot 10^{-31}$, $n = 2.6$, $k_\infty = 3.6 \cdot 10^{-11}$, $m = 0.1$, $[M] = [\text{air}]$
[h] $k_0 = 1.8 \cdot 10^{-30}$, $n = 3.0$, $k_\infty = 2.8 \cdot 10^{-11}$, $m = 0$, $[M] = [\text{air}]$
[i] $k_0 = 9.3 \cdot 10^{-32}$, $n = 3.9$, $k_\infty = 4.2 \cdot 10^{-11}$, $m = 0.5$, $[M] = [\text{air}]$
[j] $k_0 = 1.9 \cdot 10^{-31}$, $n = 3.4$, $k_\infty = 4.0 \cdot 10^{-12}$, $m = 0.3$, $[M] = [\text{air}]$
[k] $k_0 = 2.4 \cdot 10^{-30}$, $n = 3.0$, $k_\infty = 1.6 \cdot 10^{-12}$, $m = -0.1$, $[M] = [\text{air}]$

6.2 Thermal decomposition reactions

Supplementary Table 10 shows thermal decomposition reactions used in the box model. Thermal lifetimes at 298 K and the temperature at which laboratory data were collected, 283 K and 263 K, are given for reference. Reaction rate constant expressions are taken from the literature, except for the decomposition IOIO → OIO + I. Here, theory used in this study predicts IOIO to be thermally stable with regard to CLOUD timescales (Table 1, Supplementary Section 6). I_2O_3 is predicted to be thermally stable [43], but sinks for I_2O_3 might be underestimated (Supplementary Section 2.4).

Supplementary Table 10: Thermal decomposition rate expressions and lifetimes of iodine species in the box model.

Reaction	k [s^{-1}]	$t_{298\,K}$ [s]	$t_{283\,K}$ [s]	$t_{263\,K}$ [s]	notes
IOIO → OIO + I	$8.4 \cdot 10^{13} \cdot \exp(-12026\,K/T)$	$4.0 \cdot 10^3$	$3.4 \cdot 10^4$	$8.6 \cdot 10^5$	this study
IOIO → IO + IO	[a]	$3.1 \cdot 10^2$	$2.2 \cdot 10^3$	$4.4 \cdot 10^4$	[41]
I_2O_3 → OIO + IO		$1.67 \cdot 10^{11}$			[43]
I_2O_4 → OIO + OIO	[b]	$2.0 \cdot 10^1$	$1.7 \cdot 10^2$	$4.6 \cdot 10^3$	[41]
$IONO_2$ → IO + NO_2	$1.1 \cdot 10^{15} \cdot \exp(-12060\,K/T)$	$3.4 \cdot 10^2$	$2.9 \cdot 10^3$	$7.5 \cdot 10^4$	[50]

[a] $k = (2.55355 \cdot 10^{11} - 4.41888 \cdot 10^7 \cdot 0.75p/\mathrm{Pa} + 856.186 \cdot (0.75 \cdot p/\mathrm{Pa})^2 + 1.421881 \cdot 10^{-2} \cdot (0.75p/\mathrm{Pa})^3) \cdot \exp((-11466.82304 + 597.01334 \cdot \exp(-0.75 \cdot p/\mathrm{Pa}/138262.325) - 167.3391 \cdot \exp(-0.75 \cdot p/\mathrm{Pa}/4375.089))\,\mathrm{K}/T)$

[b] $k = (-1.92626 \cdot 10^{14} + 4.67414 \cdot 10^{11} \cdot 0.75p/\mathrm{Pa} - 36865.1 \cdot (0.75 \cdot p/\mathrm{Pa})^2 - 3.09109 \cdot (0.75p/\mathrm{Pa})^3) \cdot \exp((-12302.15294 + 152.78367 \cdot \exp(-0.75p/\mathrm{Pa}/4612.733) + 437.62868 \cdot \exp(-0.75 \cdot p/\mathrm{Pa}/42844.13))\,\mathrm{K}/T)$

6.3 Photochemistry

The CLOUD chamber employs different lights to selectively drive photochemistry. All lights are continuously characterised and monitored by a spectrometer and photo diode array at the bottom of the chamber, and by dedicated actinometry experiments which allow to quantitatively determine actinic fluxes. Measured spectra, scaled into units of actinic fluxes, are shown in Supplementary Fig. 10. Resulting photolysis rates and photolysis reactions are listed in Supplementary Table 11. The intensity of each light source can be regulated, such that the actinic fluxes and photolysis rates shown are upper limits. The photolysis frequencies shown in Supplementary Table 11 are derived using cross-section and quantum yield recommendations from [51], retrieved from [52]. The cross sections used for I_2O_2, I_2O_3, and I_2O_4 are those predicted by theory [18], given that attempts to measure these cross sections [19] have not been conclusive [18].

Specifically, LS4 is a *light sabre* protruding laterally into the chamber, i.e., an array of LEDs centred at 528 nm (green light). LS4 was purposefully built to selectively photolyse I_2. The ability of LS4 to photolyse I_2 was determined in actinometry experiments which inferred the actinic flux from the decay rate of I_2 (Supplementary Fig. 11). The uncertainty of j_{I_2} is estimated to be better than 30%, based on variability at different experimental conditions. The absorption cross section of I_2, in conjunction with the dissociation quantum yield is used to estimate the (spectral) actinic flux due to LS4. The quantum yield above 492 nm for dissociation is not established to be unity, but closer to 70 % in the wavelength range of overlap [53]. The uncertainty in the quantum yield is not an uncertainty for the photo dissociation rate of I_2, but for the scaling of the actinic flux. For the latter, an uncertainty of 40 % has to be assumed. Usually LS4 is not used at full power, to ensure near-homogeneous mixing within the chamber. However, at full LS4 power, photolysis can be a competitive sink for OIO (Supplementary Table 11).

UVX is a krypton fluoride laser (248 nm) and a selective source for the production of $O(^1D)$ and HO_x. UVH is a mercury lamp and provides light across the entire UV-Vis spectral range. LS3 is a blue LED

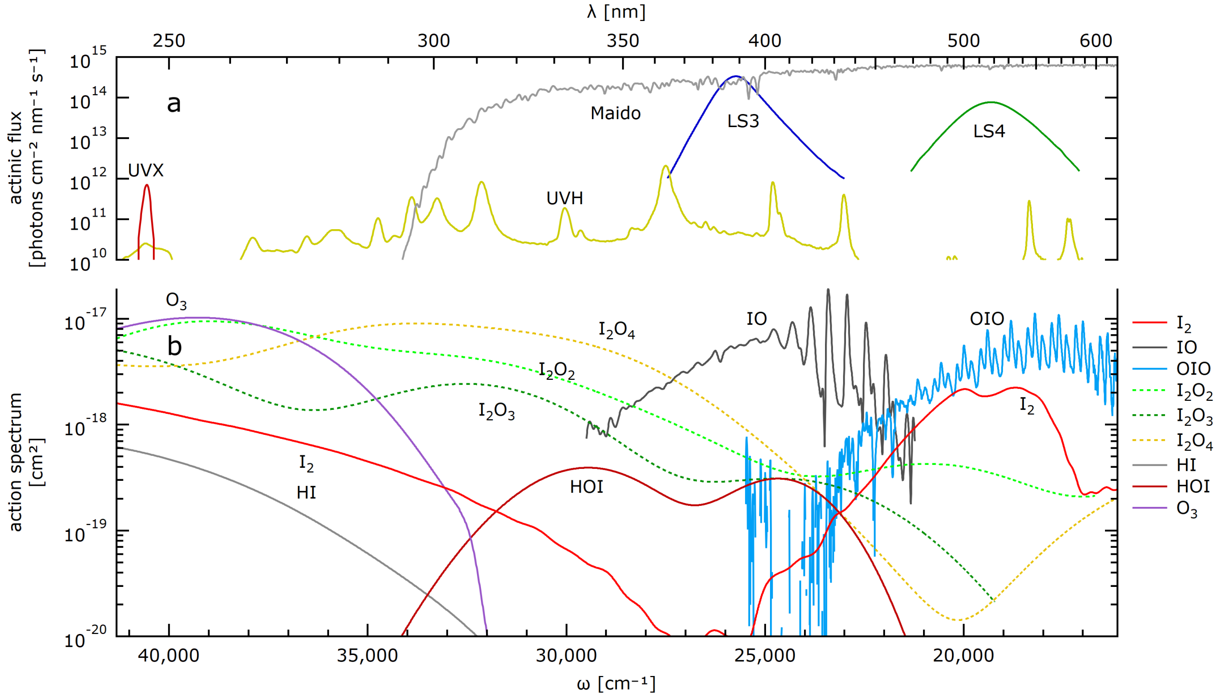

Supplementary Fig. 10: Iodine photochemistry at the CLOUD chamber. The top panel (a) shows estimates of the spectral actinic fluxes from the different light sources, and for noon-time conditions at the Maïdo field site. The bottom panel (b) shows action spectra (product of absorption cross section and total quantum yield) of some iodine species represented in the model. The cross sections of I_2O_2, I_2O_3, and I_2O_4 are predicted from theory only, i.e. not measured across the spectral range shown.

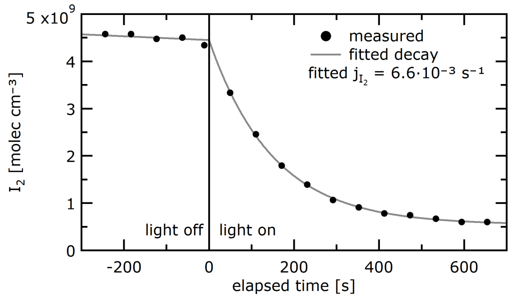

Supplementary Fig. 11: Actinometry experiment to determine photolysis frequency of I_2 due to LS4.

light source (centred at 385 nm). It is capable of photolysing IO fast, but I radicals readily recombine with O_3 to reform IO. Hence, sensitivity studies that varied illumination from LS3 did not find a sensitivity.

Supplementary Table 11: Photolysis reactions included in chemical box model with photolysis rates due to different different lamps (upper limit at maximum continuous intensity) and solar light.

Reaction	j [s^{-1}]				
	LS4 [a]	LS3 [b]	UVH [c]	UVX [c]	Maïdo [d]
$I_2 \rightarrow I + I$	$6.5 \cdot 10^{-3}$ [e]	$7.4 \cdot 10^{-5}$	$5.8 \cdot 10^{-6}$	$1.1 \cdot 10^{-6}$	$1.3 \cdot 10^{-1}$
$IO \rightarrow I + O(^3P)$		$2.9 \cdot 10^{-2}$	$5.3 \cdot 10^{-5}$		$1.9 \cdot 10^{-1}$
$OIO \rightarrow I + O_2$ [h]	$1.5 \cdot 10^{-2}$		$1.3 \cdot 10^{-5}$		$4.5 \cdot 10^{-1}$
$I_2O_2 \rightarrow IO + IO$ [fi]	$1.1 \cdot 10^{-3}$	$2.8 \cdot 10^{-3}$	$4.4 \cdot 10^{-5}$	$6.0 \cdot 10^{-5}$	$6.1 \cdot 10^{-2}$
$I_2O_3 \rightarrow OIO + IO$ [i]	$6.4 \cdot 10^{-5}$	$1.6 \cdot 10^{-3}$	$2.0 \cdot 10^{-5}$	$3.3 \cdot 10^{-6}$	$2.1 \cdot 10^{-2}$
$I_2O_4 \rightarrow OIO + OIO$ [i]	$9.3 \cdot 10^{-5}$	$5.5 \cdot 10^{-3}$	$8.6 \cdot 10^{-5}$	$2.7 \cdot 10^{-6}$	$7.3 \cdot 10^{-2}$
$HOI \rightarrow I + OH$	$7.0 \cdot 10^{-5}$	$1.3 \cdot 10^{-3}$	$3.9 \cdot 10^{-6}$		$9.0 \cdot 10^{-3}$
$HI(+O_2) \rightarrow HO_2 + I$			$3.2 \cdot 10^{-7}$	$4.0 \cdot 10^{-7}$	$1.1 \cdot 10^{-5}$
$INO \rightarrow I + NO$		$4.1 \cdot 10^{-3}$	$3.2 \cdot 10^{-5}$	$4.3 \cdot 10^{-5}$	$3.2 \cdot 10^{-2}$
$INO_2 \rightarrow I + NO_2$		$2.2 \cdot 10^{-5}$	$5.8 \cdot 10^{-6}$	$2.9 \cdot 10^{-6}$	$3.0 \cdot 10^{-3}$
$IONO_2 \rightarrow I + NO_3$		$6.8 \cdot 10^{-3}$	$6.4 \cdot 10^{-5}$	$9.1 \cdot 10^{-6}$	$4.8 \cdot 10^{-2}$
$O_3 \rightarrow O_2 + O(^1D)$			$7.0 \cdot 10^{-6}$ [e]	$7.0 \cdot 10^{-6}$ [e]	$2.7 \cdot 10^{-5}$
$H_2O_2 \rightarrow OH + OH$			$7.4 \cdot 10^{-8}$	$7.2 \cdot 10^{-8}$	$7.2 \cdot 10^{-6}$
$NO_2 \rightarrow NO + O(^3P)$		$3.0 \cdot 10^{-3}$ [e]	$7.4 \cdot 10^{-6}$	$1.8 \cdot 10^{-8}$	$9.8 \cdot 10^{-3}$
$HONO \rightarrow OH + NO$		$2.7 \cdot 10^{-4}$	$1.4 \cdot 10^{-6}$	$2.0 \cdot 10^{-7}$	$1.6 \cdot 10^{-3}$
$NO_3 \rightarrow NO_2 + O(^3P)$ [g] $\rightarrow NO + O_2$ [g]	$6.2 \cdot 10^{-3}$	$7.7 \cdot 10^{-6}$	$1.1 \cdot 10^{-5}$		$3.9 \cdot 10^{-1}$
$HNO_3 \rightarrow OH + NO_2$			$2.5 \cdot 10^{-8}$	$1.5 \cdot 10^{-8}$	$6.3 \cdot 10^{-7}$
$N_2O_4 \rightarrow NO_2 + NO_2$		$7.5 \cdot 10^{-5}$	$4.0 \cdot 10^{-6}$		$4.4 \cdot 10^{-3}$
$N_2O_5 \rightarrow NO_3 + NO_2$		$1.2 \cdot 10^{-6}$	$3.9 \cdot 10^{-7}$	$3.4 \cdot 10^{-7}$	$4.7 \cdot 10^{-5}$

[a] characterised via decay rate of I_2, uncertainty approximately 30%
[b] characterised via NO_2 : NO : O_3, uncertainty approximately 30%
[c] characterised via production of H_2SO_4
[d] actinic fluxes calculated by TUV [30]
[e] directly determined in actinometry experiments
[f] products assumed to be IO, based on thermal stability (Supplementary Table 10)
[g] quantum yields not well known, equal branching assumed
[h] using cross section of [20]
[i] using cross section predicted in [18]

References

[1] He, X.-C., Tham, Y.J., Dada, L., Wang, M., Finkenzeller, H., Stolzenburg, D., Iyer, S., Simon, M., Kürten, A., Shen, J., Rörup, B., Rissanen, M., Schobesberger, S., Baalbaki, R., Wang, D.S., Koenig, T.K., Jokinen, T., Sarnela, N., Beck, L.J., Almeida, J., Amanatidis, S., Amorim, A., Ataei, F., Baccarini, A., Bertozzi, B., Bianchi, F., Brilke, S., Caudillo, L., Chen, D., Chiu, R., Chu, B., Dias, A., Ding, A., Dommen, J., Duplissy, J., El Haddad, I., Gonzalez Carracedo, L., Granzin, M., Hansel, A., Heinritzi, M., Hofbauer, V., Junninen, H., Kangasluoma, J., Kemppainen, D., Kim, C., Kong, W., Krechmer, J.E., Kvashin, A., Laitinen, T., Lamkaddam, H., Lee, C.P., Lehtipalo, K., Leiminger, M., Li, Z., Makhmutov, V., Manninen, H.E., Marie, G., Marten, R., Mathot, S., Mauldin, R.L., Mentler, B., Möhler, O., Müller, T., Nie, W., Onnela, A., Petäjä, T., Pfeifer, J., Philippov, M., Ranjithkumar, A., Saiz-Lopez, A., Salma, I., Scholz, W., Schuchmann, S., Schulze, B., Steiner, G., Stozhkov, Y., Tauber, C., Tomé, A., Thakur, R.C., Väisänen, O., Vazquez-Pufleau, M., Wagner, A.C., Wang, Y., Weber, S.K., Winkler, P.M., Wu, Y., Xiao, M., Yan, C., Ye, Q., Ylisirniö, A., Zauner-Wieczorek, M., Zha, Q., Zhou, P., Flagan, R.C., Curtius, J., Baltensperger, U., Kulmala, M., Kerminen, V.-M., Kurtén, T., Donahue, N.M., Volkamer, R., Kirkby, J., Worsnop, D.R., Sipilä, M.: Role of iodine oxoacids in atmospheric aerosol nucleation. Science (80-.). **371**(6529), 589–595 (2021). https://doi.org/10.1126/science.abe0298

[2] Gómez Martín, J.C., Lewis, T.R., Blitz, M.A., Plane, J.M.C., Kumar, M., Francisco, J.S., Saiz-Lopez, A.: A gas-to-particle conversion mechanism helps to explain atmospheric particle formation through clustering of iodine oxides. Nat. Commun. **11**(1), 1–14 (2020). https://doi.org/10.1038/s41467-020-18252-8

[3] Dix, B., Baidar, S., Bresch, J.F., Hall, S.R., Schmidt, K.S., Wang, S., Volkamer, R.: Detection of iodine monoxide in the tropical free troposphere. Proc. Natl. Acad. Sci. **110**(6), 2035–2040 (2013). https://doi.org/10.1073/pnas.1212386110

[4] Wang, S., Schmidt, J.A., Baidar, S., Coburn, S., Dix, B., Koenig, T.K., Apel, E., Bowdalo, D., Campos, T.L., Eloranta, E.W., Evans, M.J., DiGangi, J.P., Zondlo, M.A., Gao, R.-S., Haggerty, J.A., Hall, S.R., Hornbrook, R.S., Jacob, D., Morley, B., Pierce, B., Reeves, M., Romashkin, P., ter Schure, A., Volkamer, R.: Active and widespread halogen chemistry in the tropical and subtropical free troposphere. Proc. Natl. Acad. Sci. **112**(30), 9281–9286 (2015). https://doi.org/10.1073/pnas.1505142112

[5] Volkamer, R., Baidar, S., Campos, T.L., Coburn, S.C., DiGangi, J.P., Dix, B., Eloranta, E.W., Koenig, T.K., Morley, B., Ortega, I., Pierce, B.R., Reeves, M., Sinreich, R., Wang, S., Zondlo, M.A., Romashkin, P.A.: Aircraft measurements of BrO, IO, glyoxal, NO_2, H_2O, O_2–O_2 and aerosol extinction profiles in the tropics: comparison with aircraft-/ship-based in situ and lidar measurements. Atmos. Meas. Tech. **8**(5), 2121–2148 (2015). https://doi.org/10.5194/amt-8-2121-2015

[6] Mahajan, A.S., Martín, J.C.G., Hay, T.D., Royer, S.-J., Yvon-Lewis, S., Liu, Y., Hu, L., Prados-Roman, C., Ordóñez, C., Plane, J.M.C., Saiz-Lopez, A.: Latitudinal distribution of reactive iodine in the Eastern Pacific and its link to open ocean sources. Atmos. Chem. Phys. **12**(23), 11609–11617 (2012). https://doi.org/10.5194/acp-12-11609-2012

[7] Saiz-Lopez, A., Plane, J.M.C., Baker, A.R., Carpenter, L.J., von Glasow, R., Gómez Martín, J.C., McFiggans, G., Saunders, R.W.: Atmospheric Chemistry of Iodine. Chem. Rev. **112**(3), 1773–1804 (2012). https://doi.org/10.1021/cr200029u

[8] Huang, R.-J., Seitz, K., Buxmann, J., Pöhler, D., Hornsby, K.E., Carpenter, L.J., Platt, U., Hoffmann, T.: In situ measurements of molecular iodine in the marine boundary layer: the link to

macroalgae and the implications for O_3, IO, OIO and NO_x. Atmos. Chem. Phys. **10**(10), 4823–4833 (2010). https://doi.org/10.5194/acp-10-4823-2010

[9] O'Dowd, C.D., Jimenez, J.L., Bahreini, R., Flagan, R.C., Seinfeld, J.H., Hämerl, K., Pirjola, L., Kulmala, M., Hoffmann, T.: Marine aerosol formation from biogenic iodine emissions. Nature **417**(6889), 632–636 (2002). https://doi.org/10.1038/nature00775

[10] Plane, J.M.C., Joseph, D.M., Allan, B.J., Ashworth, S.H., Francisco, J.S.: An Experimental and Theoretical Study of the Reactions OIO + NO and OIO + OH. J. Phys. Chem. A **110**(1), 93–100 (2006). https://doi.org/10.1021/jp055364y

[11] Rissanen, M.P., Mikkilä, J., Iyer, S., Hakala, J.: Multi-scheme chemical ionization inlet (MION) for fast switching of reagent ion chemistry in atmospheric pressure chemical ionization mass spectrometry (CIMS) applications. Atmos. Meas. Tech. **12**(12), 6635–6646 (2019). https://doi.org/10.5194/amt-12-6635-2019

[12] Wang, M., He, X.-C., Finkenzeller, H., Iyer, S., Chen, D., Shen, J., Simon, M., Hofbauer, V., Kirkby, J., Curtius, J., Maier, N., Kurtén, T., Worsnop, D.R., Kulmala, M., Rissanen, M., Volkamer, R., Tham, Y.J., Donahue, N.M., Sipilä, M.: Measurement of iodine species and sulfuric acid using bromide chemical ionization mass spectrometers. Atmos. Meas. Tech. **14**(6), 4187–4202 (2021). https://doi.org/10.5194/amt-14-4187-2021

[13] He, X.-C., Iyer, S., Sipilä, M., Ylisirniö, A., Peltola, M., Kontkanen, J., Baalbaki, R., Simon, M., Kürten, A., Tham, Y.J., Pesonen, J., Ahonen, L.R., Amanatidis, S., Amorim, A., Baccarini, A., Beck, L., Bianchi, F., Brilke, S., Chen, D., Chiu, R., Curtius, J., Dada, L., Dias, A., Dommen, J., Donahue, N.M., Duplissy, J., El Haddad, I., Finkenzeller, H., Fischer, L., Heinritzi, M., Hofbauer, V., Kangasluoma, J., Kim, C., Koenig, T.K., Kubečka, J., Kvashnin, A., Lamkaddam, H., Lee, C.P., Leiminger, M., Li, Z., Makhmutov, V., Xiao, M., Marten, R., Nie, W., Onnela, A., Partoll, E., Petäjä, T., Salo, V.-T., Schuchmann, S., Steiner, G., Stolzenburg, D., Stozhkov, Y., Tauber, C., Tomé, A., Väisänen, O., Vazquez-Pufleau, M., Volkamer, R., Wagner, A.C., Wang, M., Wang, Y., Wimmer, D., Winkler, P.M., Worsnop, D.R., Wu, Y., Yan, C., Ye, Q., Lehtinen, K., Nieminen, T., Manninen, H.E., Rissanen, M., Schobesberger, S., Lehtipalo, K., Baltensperger, U., Hansel, A., Kerminen, V.-M., Flagan, R.C., Kirkby, J., Kurtén, T., Kulmala, M.: Determination of the collision rate coefficient between charged iodic acid clusters and iodic acid using the appearance time method. Aerosol Sci. Technol. **55**(2), 231–242 (2021). https://doi.org/10.1080/02786826.2020.1839013

[14] Mauldin, L., Cantrell, C.A., Rissanen, M., Nowak, J.B., Lambe, A.T., Canagaratna, M.R., Yan, C., Nie, W., He, X.: HOxROx Measurements of Highly-Oxidized Multifunctional Compounds Species During CLOUD and PROPHET AMOS. In: AGU Fall Meet. Abstr., vol. 2019, pp. 51–2744 (2019)

[15] Pfeifer, J., Simon, M., Heinritzi, M., Piel, F., Weitz, L., Wang, D., Granzin, M., Müller, T., Bräkling, S., Kirkby, J., Curtius, J., Kürten, A.: Measurement of ammonia, amines and iodine compounds using protonated water cluster chemical ionization mass spectrometry. Atmos. Meas. Tech. **13**(5), 2501–2522 (2020). https://doi.org/10.5194/amt-13-2501-2020

[16] Sipilä, M., Sarnela, N., Jokinen, T., Henschel, H., Junninen, H., Kontkanen, J., Richters, S., Kangasluoma, J., Franchin, A., Peräkylä, O., Rissanen, M.P., Ehn, M., Vehkamäki, H., Kurten, T., Berndt, T., Petäjä, T., Worsnop, D., Ceburnis, D., Kerminen, V.-M., Kulmala, M., O'Dowd, C.: Molecular-scale evidence of aerosol particle formation via sequential addition of HIO3. Nature **537**(7621), 532–534 (2016). https://doi.org/10.1038/nature19314

[17] Gómez Martín, J.C., Lewis, T.R., James, A.D., Saiz-Lopez, A., Plane, J.M.C.: Insights into the

Chemistry of Iodine New Particle Formation: The Role of Iodine Oxides and the Source of Iodic Acid. J. Am. Chem. Soc. (2022). https://doi.org/10.1021/jacs.1c12957

[18] Lewis, T.R., Carlos, J., Martín, G., Blitz, M.A., Cuevas, C.A., Plane, J.M.C., Saiz-lopez, A.: Determination of the absorption cross-sections of higher order iodine oxides at 355 nm and 532 nm . Atmos. Chem. Phys. **20**(18), 1–28 (2020)

[19] Gómez Martín, J.C., Spietz, P., Burrows, J.P.: Spectroscopic studies of the I2/O3 photochemistry: Part 1: Determination of the absolute absorption cross sections of iodine oxides of atmospheric relevance. J. Photochem. Photobiol. A Chem. **176**(1-3), 15–38 (2005)

[20] Spietz, P., Gómez Martín, J.C., Burrows, J.P.: Spectroscopic studies of the I2/O3 photochemistry: Part 2. Improved spectra of iodine oxides and analysis of the IO absorption spectrum. J. Photochem. Photobiol. A Chem. **176**(1–3), 50–67 (2005). https://doi.org/10.1016/j.jphotochem.2005.08.023

[21] Gómez Martín, J.C., Gálvez, O., Baeza-Romero, M.T., Ingham, T., Plane, J.M.C., Blitz, M.A.: On the mechanism of iodine oxide particle formation. Phys. Chem. Chem. Phys. **15**(37), 15612–15622 (2013). https://doi.org/10.1039/C3CP51217G

[22] Vogt, R., Sander, R., von Glasow, R., Crutzen, P.J.: Iodine Chemistry and its Role in Halogen Activation and Ozone Loss in the Marine Boundary Layer: A Model Study. J. Atmos. Chem. **32**(3), 375–395 (1999). https://doi.org/10.1023/A:1006179901037

[23] Garland, J.A., Curtis, H.: Emission of iodine from the sea surface in the presence of ozone. J. Geophys. Res. Ocean. **86**(C4), 3183–3186 (1981)

[24] Baccarini, A., Karlsson, L., Dommen, J., Duplessis, P., Vüllers, J., Brooks, I.M., Saiz-Lopez, A., Salter, M., Tjernström, M., Baltensperger, U., Zieger, P., Schmale, J.: Frequent new particle formation over the high Arctic pack ice by enhanced iodine emissions. Nat. Commun. **11**(1), 1–11 (2020). https://doi.org/10.1038/s41467-020-18551-0

[25] Khanniche, S., Louis, F., Cantrel, L., Černušák, I.: Investigation of the Reaction Mechanism and Kinetics of Iodic Acid with OH Radical Using Quantum Chemistry. ACS Earth Sp. Chem. **1**(4), 227–235 (2017). https://doi.org/10.1021/acsearthspacechem.7b00038

[26] Vaucher, A.C., Reiher, M.: Steering orbital optimization out of local minima and saddle points toward lower energy. Journal of Chemical Theory and Computation **13**(3), 1219–1228 (2017)

[27] Grimme, S.: Supramolecular binding thermodynamics by dispersion-corrected density functional theory. Chemistry–A European Journal **18**(32), 9955–9964 (2012)

[28] Shannon, R.J., Robertson, S.H., Blitz, M.A., Seakins, P.W.: Bimolecular reactions of activated species: An analysis of problematic HC(O)C(O) chemistry. Chem. Phys. Lett. **661**, 58–64 (2016). https://doi.org/10.1016/j.cplett.2016.08.055

[29] Dix, B., Koenig, T.K., Volkamer, R.: Parameterization retrieval of trace gas volume mixing ratios from Airborne MAX-DOAS. Atmos. Meas. Tech. **9**(11), 5655–5675 (2016). https://doi.org/10.5194/amt-9-5655-2016

[30] Madronich, S.: TUV software package version 4.1 a (1993)

[31] Saiz-Lopez, A., Plane, J.M.C., Cuevas, C.A., Mahajan, A.S., Lamarque, J.F., Kinnison, D.E.: Nighttime atmospheric chemistry of iodine. Atmos. Chem. Phys. **16**(24), 15593–15604 (2016).

https://doi.org/10.5194/acp-16-15593-2016

[32] Frege, C., Bianchi, F., Molteni, U., Tröstl, J., Junninen, H., Henne, S., Sipilä, M., Herrmann, E., Rossi, M.J., Kulmala, M., Hoyle, C.R., Baltensperger, U., Dommen, J.: Chemical characterization of atmospheric ions at the high altitude research station Jungfraujoch (Switzerland). Atmos. Chem. Phys. **17**(4), 2613–2629 (2017). https://doi.org/10.5194/acp-17-2613-2017

[33] Baccarini, A., Dommen, J., Lehtipalo, K., Henning, S., Modini, R.L., Gysel-Beer, M., Baltensperger, U., Schmale, J.: Low-volatility vapors and new particle formation over the Southern Ocean during the Antarctic Circumnavigation Expedition. Earth Sp. Sci. Open Arch., 31 (2021). https://doi.org/10.1002/essoar.10506899.1

[34] De Leeuw, G., Kunz, G.J., Buzorius, G., O'Dowd, C.D.: Meteorological influences on coastal new particle formation. J. Geophys. Res. Atmos. **107**(19), 1–11 (2002). https://doi.org/10.1029/2001JD001478

[35] Saunders, R.W., Kumar, R., Gómez Martín, J.C., Mahajan, A.S., Murray, B.J., Plane, J.M.C.: Studies of the formation and growth of aerosol from molecular iodine precursor. Zeitschrift fur Phys. Chemie **224**(7-8), 1095–1117 (2010). https://doi.org/10.1524/zpch.2010.6143

[36] Jimenez, J.L., Bahreini, R., Cocker, D.R., Zhuang, H., Varutbangkul, V., Flagan, R.C., Seinfeld, J.H., O'Dowd, C.D., Hoffmann, T.: New particle formation from photooxidation of diiodomethane (CH2I2). J. Geophys. Res. Atmos. **108**(D10) (2003). https://doi.org/10.1029/2002JD002452

[37] Murray, B.J., Haddrell, A.E., Peppe, S., Davies, J.F., Reid, J.P., O'Sullivan, D., Price, H.C., Kumar, R., Saunders, R.W., Plane, J.M.C., Umo, N.S., Wilson, T.W.: Glass formation and unusual hygroscopic growth of iodic acid solution droplets with relevance for iodine mediated particle formation in the marine boundary layer. Atmos. Chem. Phys. **12**(18), 8575–8587 (2012). https://doi.org/10.5194/acp-12-8575-2012

[38] Koenig, T.K., Baidar, S., Campuzano-Jost, P., Cuevas, C.A., Dix, B., Fernandez, R.P., Guo, H., Hall, S.R., Kinnison, D., Nault, B.A., Ullmann, K., Jimenez, J.L., Saiz-Lopez, A., Volkamer, R.: Quantitative detection of iodine in the stratosphere. Proc. Natl. Acad. Sci. U. S. A. **117**(4), 1860–1866 (2020). https://doi.org/10.1073/pnas.1916828117

[39] Burkholder, J.B., Sander, S.P., Abbatt, J., Barker, J.R., Cappa, C., Crounse, J.D., Dibble, T.S., Huie, R.E., Kolb, C.E., Kurylo, M.J., Orkin, V.L., Percival, C.J., Wilmouth, D.M., Wine, P.H.: Chemical Kinetics and Photochemical Data for Use in Atmospheric Studies, Evaluation No. 19. Technical report, Jet Propulsion Laboratory, Pasadena (2019). http://jpldataeval.jpl.nasa.gov

[40] Koenig, T.K., Volkamer, R., Apel, E.C., Bresch, J.F., Cuevas, C.A., Dix, B., Eloranta, E.W., Fernandez, R.P., Hall, S.R., Hornbrook, R.S., Pierce, R.B., Reeves, J.M., Saiz-Lopez, A., Ullmann, K.: Ozone depletion due to dust release of iodine in the free troposphere. Sci. Adv. **7**(52), 6544 (2021). https://doi.org/10.1126/sciadv.abj6544

[41] Saiz-Lopez, A., Fernandez, R.P., Ordonez, C., Kinnison, D.E., Gomez Martin, J.C., Lamarque, J.-F., Tilmes, S.: Iodine chemistry in the troposphere and its effect on ozone. Atmos. Chem. Phys. **14**(23), 13119–13143 (2014). https://doi.org/10.5194/acp-14-13119-2014

[42] Vikis, A.C., MacFarlane, R.: Reaction of iodine with ozone in the gas phase. J. Phys. Chem. **89**(5), 812–815 (1985). https://doi.org/10.1021/j100251a019

[43] Galvez, O., Gomez Martín, J.C., Gomez, P.C., Saiz-Lopez, A., Pacios, L.F.: A theoretical study

on the formation of iodine oxide aggregates and monohydrates. Phys. Chem. Chem. Phys. **15**(37), 15572–15583 (2013). https://doi.org/10.1039/C3CP51219C

[44] Dillon, T.J., Tucceri, M.E., Crowley, J.N.: Laser induced fluorescence studies of iodine oxide chemistry Part II. The reactions of IO with CH3O2, CF3O2 and O3. Phys. Chem. Chem. Phys. **8**(44), 5185–5198 (2006). https://doi.org/10.1039/B611116E

[45] McFiggans, G., Plane, J.M.C., Allan, B.J., Carpenter, L.J., Coe, H., O'Dowd, C.: A modeling study of iodine chemistry in the marine boundary layer. J. Geophys. Res. Atmos. **105**(D11), 14371–14385 (2000). https://doi.org/10.1029/1999JD901187

[46] Chameides, W.L., Davis, D.D.: Iodine: Its possible role in tropospheric photochemistry. J. Geophys. Res. Ocean. **85**(C12), 7383–7398 (1980). https://doi.org/10.1029/JC085iC12p07383

[47] Bösch, H., Camy-Peyret, C., Chipperfield, M.P., Fitzenberger, R., Harder, H., Platt, U., Pfeilsticker, K.: Upper limits of stratospheric IO and OIO inferred from center-to-limb-darkening-corrected balloon-borne solar occultation visible spectra: Implications for total gaseous iodine and stratospheric ozone. J. Geophys. Res. Atmos. **108**(D15) (2003). https://doi.org/10.1029/2002JD003078

[48] Chambers, R.M., Heard, A.C., Wayne, R.P.: Inorganic gas-phase reactions of the nitrate radical: iodine+ nitrate radical and iodine atom+ nitrate radical. J. Phys. Chem. **96**(8), 3321–3331 (1992)

[49] Kaltsoyannis, N., Plane, J.M.C.: Quantum chemical calculations on a selection of iodine-containing species (IO, OIO, INO3, (IO)2, I2O3, I2O4 and I2O5) of importance in the atmosphere. Phys. Chem. Chem. Phys. **10**(13), 1723–1733 (2008). https://doi.org/10.1039/B715687C

[50] Allan, B.J., Plane, J.M.C.: A Study of the Recombination of IO with NO2 and the Stability of INO3: Implications for the Atmospheric Chemistry of Iodine. J. Phys. Chem. A **106**(37), 8634–8641 (2002). https://doi.org/10.1021/jp020089q

[51] Sander, S.P., Abbatt, J., Barker, J.R., Burkholder, J.B., Friedl, R.R., Golden, D.M., Huie, R.E., Kolb, C.E., Kurylo, M.J., Moortgat, G.K., Others: Chemical kinetics and photochemical data for use in atmospheric studies, evaluation no. 17, JPL Publication 10-6. Jet Propuls. Lab. Pasadena **15** (2011)

[52] Keller-Rudek, H., Moortgat, G.K., Sander, R., Sörensen, R.: The MPI-Mainz UV/VIS Spectral Atlas of Gaseous Molecules of Atmospheric Interest. Earth Syst. Sci. Data **5**(2), 365–373 (2013). https://doi.org/10.5194/essd-5-365-2013

[53] Brewer, L., Tellinghuisen, J.: Quantum Yield for Unimolecular Dissociation of I2 in Visible Absorption. J. Chem. Phys. **56**(8), 3929–3938 (1972). https://doi.org/10.1063/1.1677797
